# Bayesian splines versus fractional polynomials in network meta-analysis

**DOI:** 10.1186/s12874-020-01113-9

**Published:** 2020-10-20

**Authors:** Andreas Heinecke, Marta Tallarita, Maria De Iorio

**Affiliations:** 1 Yale-NUS College, 16 College Avenue West, Singapore, 138527 Singapore; 2 Department of Statistical Science, University College London, Gower Street, London, WC1E 6BT UK

**Keywords:** Bayesian evidence synthesis techniques, P-splines, Clinical trials, Evidence-synthesis, Longitudinal studies, Markov chain Monte Carlo methods, Mixed treatment comparison

## Abstract

**Background:**

Network meta-analysis (NMA) provides a powerful tool for the simultaneous evaluation of multiple treatments by combining evidence from different studies, allowing for direct and indirect comparisons between treatments. In recent years, NMA is becoming increasingly popular in the medical literature and underlying statistical methodologies are evolving both in the frequentist and Bayesian framework. Traditional NMA models are often based on the comparison of two treatment arms per study. These individual studies may measure outcomes at multiple time points that are not necessarily homogeneous across studies.

**Methods:**

In this article we present a Bayesian model based on B-splines for the simultaneous analysis of outcomes across time points, that allows for indirect comparison of treatments across different longitudinal studies.

**Results:**

We illustrate the proposed approach in simulations as well as on real data examples available in the literature and compare it with a model based on P-splines and one based on fractional polynomials, showing that our approach is flexible and overcomes the limitations of the latter.

**Conclusions:**

The proposed approach is computationally efficient and able to accommodate a large class of temporal treatment effect patterns, allowing for direct and indirect comparisons of widely varying shapes of longitudinal profiles.

## Background

Scientific and technological advances are steadily adding to the number of different healthcare interventions. To fully exploit their potential requires clinicians and healthcare professionals to make informed and objective choices, based on clinical studies, between a possibly large number of treatment options in terms of relative medical efficacy and cost effectiveness [11, 22]. It is generally accepted that randomized controlled trials provide the most rigorous and conclusive evidence on the relative effects of different interventions. For example, the gold standard for directly comparing two treatments *A* and *B* is a randomized controlled trial. In practice, however, evidence from direct comparison trials may be limited and it is often impossible to have head-to-head comparisons for all relevant comparators of an intervention, making it necessary to resort to indirect comparisons [11]. For instance, direct comparison from two different studies on treatment *A* versus *C*, and *B* versus *C*, might be available and indirect methods exploit the common comparator *C* to provide an indirect comparison of treatment *A* versus *B*. A large number of individual studies can be mapped out as a network in which treatments are represented by nodes that are connected by edges where data from direct head-to-head studies comparing them are available. Network meta-analysis (NMA) refers to methods attempting to systematically integrate all the information provided by such networks of studies via the entirety of paths between different treatments. The goal is to provide a comparison of each treatment against a common comparator chosen from the network, such as a placebo or standard treatment, which consequently allows for a relative comparison among all available treatments [33, 40–43]. Several statistical methods are available in the literature to effectively integrate findings from individual studies and implement NMA in the context of systematic reviews. Current approaches include the adjusted indirect comparison method with aggregate data, meta-regression, mixed effect models, hierarchical models and Bayesian methods [43–45], and have the potential to yield useful information for clinical decision-making. The main methodological idea beyond most statistical methods is to extend standard pairwise meta-analysis techniques to the entire set of trials and to include direct and indirect comparisons by exploiting the different paths defined on the network. In the recent literature the Bayesian approach has proved successful in addressing the major statistical challenges associated to the design of a systematic review. A summary of the most recurring challenges encountered in NMA is presented in [41]: the total number of trials in a network, the number of trials with more than two comparison arms (introducing an additional layer of complexity) [1], heterogeneity (i.e. clinical, methodological, and statistical variability within direct and indirect comparisons, and across studies), inconsistency (i.e. discrepancy between direct and indirect comparisons) [2], and potential bias that may influence effect estimates [3].

In this work, we focus on NMA techniques for longitudinal data, i.e. on the NMA of studies in which individuals are assessed at multiple time points throughout the follow-up period. Among the additional difficulties this setup brings about are that follow-up times may vary across studies, and, moreover, that the repeated measurements for each individual during the follow-up tend to be correlated. The latter needs to be taken into account to avoid biased estimates of treatment effects. For instance, in [4] the authors compare alternative approaches to handling correlation in linear mixed effect models for longitudinal NMA. In a Bayesian framework [5] proposes a model based on piecewise exponential hazards, while in [6] the treatment effect in multi-arm trials is modelled with a piecewise linear function. Our starting point is the review paper [7], which compares three recently popular NMA methods for longitudinal data, whose main inferential focus is treatment effect over time and which allow for a large class of temporal patterns. The three methods are (i) the mixed treatment comparison (MTC) developed in [8], which assumes random relative effects and allows for the relative treatment effects to vary over time, without temporal pattern restriction, (ii) the Bayesian evidence synthesis techniques – integrated two-component prediction (BEST-ITP) proposed in [9], a parametric model to describe a non-linear relationship between outcome and time for each treatment, assuming a diminishing return time course of treatment responses, and (iii) the more recent method based on fractional polynomial (FP) temporal patterns of [10], which we will review in more detail below. Each of the three models is formulated under a Bayesian hierarchical modelling approach, which provides a natural tool to combine different (and possibly not completely homogeneous) sources of evidence by decomposing a complex problem in subcomponents which are modelled individually and then linked together via hyper-parameters in a probabilistically sound way. Moreover, using a Bayesian approach, it is in principle straightforward to incorporate decision making, for example allowing quantification of the impact of model- and parameter-uncertainty on the optimal decision, given current data. This process is required by many regulatory agencies (see [12]) and would simply translate in adding extra layers to the model hierarchy (see [13, 14]).

The authors of [7] conclude, based on a simulation study and real data applications, that the MTC method appears to be most conservative in the estimation of the underlying effect-size, that the BEST-ITP model fails to capture constant treatment effect over time, and that the FP model seems to offer the most flexible strategy, accommodating different time patterns. In general, it is challenging to capture non-monotonic temporal patterns, with the FP model leading to slightly more accurate estimates (as shown in their simulation study). In terms of computational cost, there are no substantial differences in running time between the three different models, although fractional polynomials require extensive sensitivity analysis to select the optimal order and power terms, as we will discuss below.

Although the FP model turns out as the best of the three reviewed approaches, it still presents limitations both from a methodological and a computational point of view (see Sections on the FP method and “Real data application” section below). In this work, we propose an NMA method based on B-splines to model temporal behaviour, which allows for the simultaneous analysis of outcomes at different time points, automatically accounting for correlation across time. We illustrate the model in simulations and on real data examples and compare its performance to the FP model. We also consider a variation of our basic model based on P-splines, which provides substantially identical inferential results. The proposed approach has many advantages in terms of model flexibility, computational burden and ease of specification. B-splines (and their extensions such a P-splines) are a natural competitor of FPs and as such they have been previously compared in the literature in different setups (see, among others, [15]).

The article is organized as follows. In the “Method” section, we describe the model likelihood for the general NMA problem. We review the FP approach, introduce the proposed B-spline model and highlight its advantages. Further, we specify the prior distribution for the parameters of the model and introduce the P-spline alternative. We then briefly describe the MCMC strategy. In the “Results” and “Discussion” sections we compare the B-spline, P-spline and FP models in simulations and real data applications. In Additional file 1, we describe in detail additional simulation scenarios and their results. We provide JAGS code to fit the proposed approach in Additional file 3.

## Method

### Network meta-analysis

The main objective of NMA of longitudinal studies is the evaluation of a suitably defined response over time. The response *y*_*sjt*_ in study *s*∈{1,…,*S*} and treatment arm *j*∈{1,…,*J*} at time *t*∈[0,*T*] is distributed as

1 $$\begin{array}{*{20}l} y_{{sjt}} \sim f\left(y_{{sjt}}\mid \theta_{{sjt}}, \sigma_{{sjt}}^{2}\right), \end{array} $$

where *f* is a (usually parametric) probability density. The main parameter of clinical interest is *θ*_*sjt*_, measuring the treatment effect of intervention *j* in study *s* at time *t*. As in the generalized linear model framework, a link function *g*(*θ*_*sjt*_) specifies the relationship between the main clinical effects *θ*_*sjt*_ and the response. For example, in the case of continuous outcomes we consider

2 $$\begin{array}{*{20}l} y_{{sjt}} \sim \text{Normal} \left(\theta_{{sjt}}, \sigma_{{sjt}}^{2}\right), \end{array} $$

with *g*(*θ*_*sjt*_)=*θ*_*sjt*_, while in the case of binary outcomes

3 $$\begin{array}{*{20}l} &y_{{sjt}} \sim \text{Binomial} \left(n_{{sjt}}, p_{{sjt}}\right),\\ &g\left(p_{{sjt}}\right)=\text{logit}\left(p_{{sjt}}\right)=\theta_{{sjt}}.  \end{array} $$

Other alternatives include *g*(*θ*_*sjt*_)= ln(*θ*_*sjt*_) if *f* is modelled as a Poisson distribution. In this work, we focus on continuous and binary responses as these are the most common outcomes in clinical trials.

The fundamental difference between the NMA methods compared in the review paper [7] is the way the treatment effect *θ*_*sjt*_ is modelled. The review shows that among the considered models FP methods are the most flexible and can accommodate a variety of treatment effect patterns. On the other hand, they require intensive computations for the choice of parameters and present modelling drawbacks, as discussed in the next Section.

To capture the longitudinal data, in this work we focus on basis function models which do not require a priori assumptions on the temporal pattern of the treatment effect. We model the longitudinal curve as a linear combination of basis functions

$$\theta_{{sjt}}=\sum\limits_{k=0}^{M}\beta_{{ksj}}h_{k}(t), $$

where *h*_*k*_ denotes a basis function, *β*_*ksj*_ is the corresponding coefficient for study *s* and treatment *j*, and *M*+1 is the total number of basis functions. In particular, we consider spline and fractional polynomial basis functions, carefully discussing the choice of the type and number of basis functions in the following sections. They are key features of the model as they influence the level of complexity and class of temporal profiles that can be accommodated.

Following [10], we set the variances of the main outcomes in (2) equal to

4 $$\begin{array}{*{20}l} \sigma^{2}_{{sjt}}= \frac{1}{\left(1-\rho^{2}\right)}\left(\frac{\text{sd}_{{sjt}}}{\sqrt{n_{{sjt}}}}\right)^{2}, \end{array} $$

i.e. to the standard error (with sd_*sjt*_ an estimate of the standard deviation and *n*_*sjt*_ the sample size) for the corresponding study, treatment arm and time point, adjusted by a factor *ρ*∈[0,1) taking into account the within-study correlation between subsequent time points. While estimates for the correlation coefficient, here assumed constant over time, may be available from expert knowledge, in a fully Bayesian setting it is an object of inference and assigned an appropriate prior distribution. In this study we assume *ρ*∼Uniform(0,0.95).

### Fractional polynomial model

In the FP regression framework, powers of the covariates of interest (in our case time), usually chosen from the set *S*={−2,−1,−0.5,0,0.5,1,2,3}, are entered into the linear predictor (see [16] for a detailed account). The authors of [10] propose an FP approach to NMA in which, given an order *M*_*F*_ and powers $\phantom {\dot {i}\!}p_{1},\ldots,p_{M_{F}}\in S$, the mean outcome of the *j*-th treatment in study *s* at time *t*>0 is modelled as

5 $$ \begin{aligned} \theta_{{sjt}}= \left\{\begin{array}{ll} \beta_{0sj}+ \sum\limits_{m=1}^{M_{F}}\beta_{{msj}}t^{p_{m}}&\text{if} p_{1}\ne \cdots \ne p_{M_{F}}, \\ \beta_{0sj}+\beta_{1sj}t^{p}+ \sum\limits_{m=2}^{M_{F}}\beta_{{msj}}t^{p}\left[\ln(t)\right]^{m-1}&\text{if} p_{1}= \cdots = p_{M_{F}} = p,\\ & \text{ and }M_{F}>1, \end{array}\right. \end{aligned}  $$

where *t*^0^:= ln(*t*). Implementing the FP model requires selecting an order *M*_*F*_ and powers $\phantom {\dot {i}\!}p_{1},\ldots,p_{M_{F}}$. In both the original work [10] and the simulation study [7] the Deviance Information Criterion [DIC, [17]] is used as an expected predictive error estimate to guide the selection of the FP order and powers. When using DIC, the aim is to select order and powers that minimize the sum of a goodness of fit term, given by the posterior mean of the model deviance, and a regularization term, given by the posterior estimate of the effective number of parameters. The latter is intended to penalize model complexity in order for the DIC to be a tool that prevents overfitting.

First and second order FPs allow representations of a considerable range of non-linear relationships, and higher orders are rarely used in medical applications (see, for instance, [18, 19]). Even considering only the cases *M*_*F*_=1 and 2, using the DIC often presents a non-negligible computational task as it requires the comparison of the DIC values of eight different first-order fractional polynomial models (one for each possible power from *S*) and of 64 different second-order fractional polynomial models (for all the possible combinations of powers from *S*). Besides the computational cost necessary to select the order and powers, the FP modelling approach lends itself to more fundamental criticism: (i) any choice of order and combination of powers imposes strong structural properties on the curve and therefore on the set of representable temporal patterns, which may not be supported by the data. Moreover, the selection of powers and order depends also on the prior specification of the other parameters in the model; (ii) FPs generally have singularities at zero or grow polynomially for large arguments. Consequently, more complex FPs may only provide poor fit for values of *t* that are large or close to zero; (iii) the individual terms in an FP are functions with support over the entire time interval and, as discussed in [16], may be less responsive locally to perturbations of the response at a given point, while the fit at distant points may be affected considerably. To avoid such effects, basis functions with short support may be more appropriate in many situations [see the discussion in [19]]; (iv) first-order FPs describe effects as a function of transformed time *t* in a linear model *β*_0*sj*_+*β*_1*sj*_*t*^*p*^, in particular they are always monotonic. Higher order FPs become increasingly complex, with second-order FPs being either monotonic or unimodal. It may however be preferable to have a model in which the linear model is always subsumed in more complex models; (v) although a second order FP as in (5) has three coefficients only (two in the case of modelling change from baseline, where the intercept is equal to zero and can be discarded), the choice of powers increases model complexity, since many different FP models need to be fitted to find the one that best describes the temporal pattern of the treatment effect. Additionally, the use of DIC as model choice criterion effectively hinges the FP model to any potential shortcomings of the DIC which have been pointed out in the literature [e.g. [20, 21]]. We have indeed observed sub-optimal DIC-based selections in the real data applications, and therefore present a more detailed discussion in the “Real data application” section. To address the above issues we next propose to use B-spline basis functions to capture the treatment effect over time.

### B-spline model

B-splines are a family of basis functions with many desirable theoretical and computational properties, making them widely used in function approximation and countless applications in statistics and engineering. For details we refer to [23]. Univariate (cardinal) B-splines can be defined inductively, starting from the B-spline of order 1 given by

$$B_{1}(t):= \left\{\begin{array}{cc} 1&\text{if} t\in[0,1],\\ 0&\text{otherwise}. \end{array}\right. $$

B-splines of higher order $n\in {\mathbb {N}}$ are defined via consecutive convolutions by

$$B_{n}(t) := {\int\nolimits}_{{\mathbb{R}}} B_{n-1}(t-s)B_{1}(t)ds, $$

i.e. with higher order they become increasingly smoother. For *n*≥2, the B-spline *B*_*n*_ is

- symmetric and positive valued, with finite support [0,*n*],
- (*n*−2)-times continuously differentiable,
- polynomial of degree *n*−1 when restricted to any interval $[k,k+1], k\in {\mathbb {Z}}$, and
- satisfies $\sum _{k\in {\mathbb {Z}}}B_{n}(t-k)=1$ for all $t\in {\mathbb {R}}$.

The integer translates $\left \{B_{n}(\cdot -k)\right \}_{k\in {\mathbb {Z}}}$ are thus a set of highly regular and well structured basis functions. They span the space of functions that are polynomial on each interval $[k,k+1], k\in {\mathbb {Z}}$, and (*n*−2)-times continuously differentiable in every $k\in {\mathbb {Z}}$. Such piecewise polynomial functions with global smoothness restrictions are called splines. In the cardinal case the integers are called the knots and the above can be easily generalized to any uniform knot sequence $(hk)_{k\in {\mathbb {Z}}}$, where $h\in {\mathbb {R}}$, by considering the basis functions $\left \{B_{n}\left (t/h-k\right)\right \}_{k\in {\mathbb {Z}}}$ of translates of the appropriately dilated cardinal B-splines. In applications in which a finite time interval is considered, only those finitely many basis functions whose support intersects the interval are required.

A main advantage of B-spline basis functions comes from their locality due to their finite support and their structure as translates of only one generating symmetric function. Choosing an order and adjacent knot distance does not impose any overall temporal behavior beyond smoothness. In fact, for sufficiently large $m\in {\mathbb {N}}$, the functions $\{B_{n}\left (2^{m}t-k\right)\}_{k\in {\mathbb {Z}}}$ can approximate any integrable function to arbitrary precision. This has to be contrasted with the FP model, in which the fractional monomials $\phantom {\dot {i}\!}t^{p_{m}}$ may be considered as basis functions, and where choosing any combination of fractional monomials imposes rather strong geometrical restrictions on the representable functions. Furthermore, due to the translation structure of the B-spline bases, all parts of the observation time-interval are treated equally, with no performance deterioration for small or large time arguments. The local support of the basis functions makes the B-spline expansions a local-influence model (i.e. perturbations of the response only affect the fit of the model locally), whereas their overlapping support, the size of which goes hand in hand with the order of smoothness, acts as a regularizing mechanism that can facilitate stability [see [16]]. B-spline bases are also stable in the sense that small changes in coefficients do not perturb the represented function significantly [see [24]]. Small changes in a represented function will therefore result in small changes in its coefficient sequence and vice versa. Together with the locality, and the partition of unity property (iv), these characteristics contribute to a better interpretability of the coefficients in a B-spline expansion as compared to the FP coefficients. Indeed, it is easy to understand changes in the function due to changes in the coefficients, as every coefficient corresponds to a particular interval of the domain of the function. On the other hand, FPs are sums of different global functions and it is therefore harder to visualize the effect of changes in coefficients. Finally, B-spline bases have the property that, with increasing order and additional knots, the spaces of representable functions become strictly larger, containing all previously representable function. This nestedness is desirable since it ensures that simpler models are always embedded in more complex, a property not shared by the FP model.

Practical applications of B-splines are dominated by *n*=4 as the order of choice, resulting in piecewise cubic polynomials with continuous curvature. In fact, given a number of data points, it is well known [e.g. [25]] that among all smooth regression functions, the unique solution to minimizing a weighted sum of squared approximation errors and the integral squared curvature as regularization is given by a cubic spline with knots at the data time-points. As such, in the remainder of this paper, we will restrict our attention to cubic B-splines.

The smoothness property of the spline functions at their knots makes them relatively insensitive to the precise locations of the knots. While observation times differ across studies, the B-spline basis functions, and in particular their knot positions, have to coincide across studies in order to guarantee the consistency assumption of the NMA in this modelling framework (see next Section). Since studies in NMA of longitudinal data typically have very few observation time points in the interval [0,*T*], we choose *h*=3/*T*, i.e. we consider the four uniformly spaced knots 0,*T*/3,2*T*/3,*T*, over the observation interval [0,*T*]. We conducted a sensitivity analysis for the number of knots ranging from two to ten and generally four knots provided the best choice (results not shown).

For cubic B-splines with four knots over the observation time interval, six of the generator translates have support intersecting [0,*T*]. We denote those by $\left \{B_{k}\right \}_{k=0}^{M_{B}}$ with *M*_*B*_=5 and model the mean outcome of treatment *j* in study *s* at time *t*>0 as cubic spline using the basis expansion

6 $$\begin{array}{*{20}l} \theta_{{sjt}}= \sum\limits_{k=0}^{M_{B}} \beta_{{ksj}} B_{k}(t). \end{array} $$

Despite having a greater number of coefficients, the B-spline model provides a conceptually simpler and computationally more attractive strategy than the FP model (5) since all order and power parameter choices (for instance evaluated via the DIC) have to be additionally considered for (5), whereas we keep the model (6), i.e. the spline order and number of knots, fixed in all applications. The localized support of B-splines allows us to propose a default model choice, with good performance in most applications. This strategy is not possible for FPs, as they lack this property and impose global assumptions on the behaviour of representable functions.

### Prior specification

We follow the prior specification proposed for the FP approach in [10], which extends earlier approaches of [8] and [9]. The regression coefficient vector $\boldsymbol {\beta }_{{sj}}=\left (\beta _{0sj}, \dots, \beta _{{Msj}}\right)^{\intercal }$ for both the FP model (5), with *M*=*M*_*F*_, and the B-spline model (6), with *M*=*M*_*B*_, is expressed as a sum of a study-specific random effect and a study-specific arm deviation from the reference treatment:

$$\begin{array}{*{20}l} \boldsymbol{\beta}_{{sj}}=\boldsymbol{\mu}_{s}+\boldsymbol{\delta}_{{sj}} \end{array} $$

where the study specific means $\boldsymbol {\mu }_{s}=\left (\mu _{0s}, \dots, \mu _{{Ms}}\right)^{\intercal }$ in our implementation are assigned a vague prior distribution

$$\begin{array}{*{20}l} \boldsymbol{\mu}_{s} \sim \text{Normal}\left(\boldsymbol{0},10^{4} \boldsymbol{I} \right), \end{array} $$

with ***0*** denoting the zero vector and ***I*** the identity matrix of appropriate dimensions. The study specific treatment effects $\boldsymbol {\delta }_{{sj}}=\left (\delta _{0sj}, \ldots, \delta _{{Msj}}\right)^{\intercal }$ in study *s* of treatment *j* relative to a reference treatment, indexed as *j*=1, are modelled as “structured random effects”

7 $$\begin{array}{*{20}l} \boldsymbol{\delta}_{{sj}} &\sim \text{Normal} \left (\boldsymbol{d}_{j}-\boldsymbol{d}_{1}, \mathbf{\Sigma}\right),  \end{array} $$

8 $$\begin{array}{*{20}l} \boldsymbol{d}_{j}&\sim\text{Normal} \left(\boldsymbol{0},10^{4} \boldsymbol{I}\right),  \end{array} $$

for *j*>1, with $\boldsymbol {d}_{1}=\left (d_{01},\ldots,d_{M1}\right)^{\intercal }:=\boldsymbol {0}$ and $\boldsymbol {\delta }_{s1}=\left (\delta _{0s1},\ldots,\delta _{Ms1}\right)^{\intercal }:=\boldsymbol {0}$. Clinical effects in (5) and (6) are therefore expressed as change from baseline (CFB) with respect to the reference treatment. The covariance matrix **Σ**=(*σ*_*m*_*σ*_*n*_*λ*_*mn*_)_*m,n*=0,…,*M*_ captures heterogeneity between studies, where $\sigma _{m}^{2}$ (often referred to as the heterogeneity parameter) represents the variance in (*δ*_*msj*_)_*sj*_ and captures how much variation exists between the results of different studies. We assume *σ*_*m*_ to be constant for all treatment comparisons. This assumption also implies that the between-study variance over effect estimates remain constant over time [see [26]]. The covariances *λ*_*mn*_ quantify the correlation between these treatment effect parameters.

When assuming a (partially) fixed-effects model, (7) (or certain components of it) is replaced by ***δ***_*sj*_=***d***_*j*_−***d***_1_ (i.e. all or some *σ*_*m*_ are zero) and it is not necessary to estimate (the respective) between-study covariances. However, in the B-spline case of this work we only consider models including random effects ***μ***_*s*_ per study, allowing for heterogeneity in the regression coefficients between studies, but fixed effects for ***δ***_*sj*_. For the FP model, as suggested in [10], we impose a random effect only on one of the components ***δ***_*sj*_. In particular, when *M*_*F*_=1, there is only one between-study heterogeneity parameter, related to the relative treatment effects for *β*_1*sj*_. In this case we have only one heterogeneity parameter *σ*_*m*_ to which we assign a uniform distribution on the interval (0,10). For all real data applications we also consider a uniform distribution on (0,5) and (0,50) for *σ*_*m*_. Posterior inference results are essentially identical to the ones obtained for the interval (0,10), showing that inference is robust to prior specification for the between study heterogenity. Moreover, if *M*_*F*_=2, we consider also the case in which the between-study heterogeneity concerns treatment effects in terms of *β*_2*sj*_.

Note that for the Bayesian NMA model described here, i.e. for (7), to facilitate consistent comparisons of treatments that are not directly compared by the same study with those that are, the implicit assumption regarding the relative treatment effects is that $d_{j_{1}}-d_{j_{2}}=\left (d_{j_{1}}-d_{1}\right) - \left (d_{j_{2}}-d_{1}\right)$ [10]. Inconsistency can occur if there are systematic differences in relative treatment effect modifiers between different direct comparisons. For the consistency assumption to transfer through (6) and (5), the same B-splines (i.e. order and knots) and the same FPs (i.e. order and powers) have to be used across all studies and treatment arms. Finally, note that the described model does not account for correlations stemming from trials with more than two treatment arms but can be easily extended to consider them [see [26]].

### Bayesian p-splines

A possible variation to the B-spline model that arises from a different prior specification for (8) is related to the penalized spline (P-spline) least squares curve fitting approach [27–29], in which B-splines with a relatively large number of equally spaced knots are used to fit a function of desired degree of smoothness by penalizing the curvature. The frequently used regularization introduced in [27] penalizes the sum of second-order differences between consecutive coefficients in the B-spline expansion, and can be adapted to incorporate other potentially available information about the shape and degree of smoothness of the target function. Most importantly, in the P-spline approach the number and location of the knots are not crucial: A relatively large number of uniformly spaced knots (large enough to ensure sufficient flexibility thus preventing oversmoothing) can be fixed a priori since the penalty term prevents overfitting. For a more thorough discussion on the P-spline penalized least squares approach see [30].

We implement a Bayesian version of the P-spline approach that has been introduced in [31]. The specification of appropriate prior distributions in (9) and (10) introduces locally adaptive smoothing parameters. As before, we assume model (6) for the treatment effects, however, the prior on ***d***_*j*_ in (8) is now replaced by a second order random walk. This provides a Bayesian counterpart to the second order penalty in [27]. Precisely, the prior specification on the ***d***_*j*_ are as follows: we set ***d***_1_=***0***, and for *j*>1 and *k*≥0 we assume, following [31],

9 $$\begin{array}{*{20}l} d_{{kj}} & \sim\text{Normal}\left(2d_{k-1,j}-d_{k-2,j},\eta_{j}\right)  \end{array} $$

10 $$\begin{array}{*{20}l} \eta_{j} & \sim \textrm{InverseGamma}(1,0.0005)  \end{array} $$

In case *d*_*k*−1,*j*_ or *d*_*k*−2,*j*_ are not available, we let *d*_*kj*_∼Normal(0,1000).

### MCMC algorithm

Posterior inference for all simulated examples and real data applications is performed via standard Gibbs sampling, implemented in JAGS [see [32]]. The first 5000 iterations are discarded as ‘burn-in’ and the final sample size on which inference is based is 10000 samples. Convergence of the chain is checked through the Gelman-Rubin potential scale reduction factor [see [34]]. The Gelman-Rubin diagnostic is evaluated by running multiple chains from different initial values and comparing the estimated between-chains and within-chain variances for each model parameter. Large differences between these variances would indicate that convergence has not been reached yet. MCMC convergence is not a major issue in this model since most prior distributions are conjugate and therefore the chain mixes well. Moreover, we are working with aggregate data that in general exhibits less variability than individual level data. In Additional file 2, we show the traceplots of ***μ***_*s*_ and ***d***_*s*_ for one of the replicas of the non-monotonic scenario for the B-spline model. Indeed, good mixing of the chain is also evident from Table 1 in which we report summary statistics of some convergence diagnostics for the non-monotonic scenario with continuous and binary responses. We note that all diagnostics are satisfactory, with the Gelman-Rubin diagnostic close to one, the Effective Sample Size close to 10000 and small MCMC standard error. JAGS code to fit the models is provided in Additional file 3.

**Table 1:** Median, minimum and maximum evaluated over the 50 replicates of the average across all the parameters of the convergence diagnostics: Gelman-Rubin dignostic, effective sample size (ESS) and Monte Carlo standard error (SE). Results are reported for our model applied to the non-monotonic scenario with binary and continuous responses

	Non-monotonic			Non-monotonic (binary)		
	Median	Minimum	Maximum	Median	Minimum	Maximum
Gelman-Rubin	1.005	1.002	1.008	1.008	1.003	1.010
ESS	9996.289	9993.762	9998.571	9114.788	9076.123	9480.754
Monte Carlo SE	0.015	0.009	0.023	0.017	0.012	0.019

## Results

### Simulation study

We have replicated the simulation study of [7] to compare the B-spline and P-spline model with the FP approach. In [7] various simulation scenarios are designed for continuous and binary responses that involve widely differing temporal patterns of treatment effects. Moreover, the authors consider data sets simulated from the models of [8] and [9], as well as simulated data from non-closed networks. In this context, a network is called closed if for all possible pairs of treatments there is at least one study providing direct comparisons. A detailed description of the simulation study setup and our results is contained in Additional file 1. Moreover, in Additional file 4 we provide R code to simulate data from all the considered scenarios. Here we only discuss one scenario in detail, which highlights the advantages of our proposed approach.

For each scenario of the simulation study, we fit the B-spline, P-spline and FP models to 50 randomly generated data sets and report results as averages. The data sets are generated as follows. Given a network structure with studies *s*, treatments *j* and observation times *t*, individual level observations for continuous outcomes are modelled by $Z_{{isjt}} \sim \text {Normal} \left (\theta _{{sjt}}, \tau _{s}^{2}\right)$, for $i=1, \dots, n_{s}$, where *n*_*s*_ is the number of observations in study *s*. The mean outcomes *θ*_*sjt*_=*γ*_*jt*_+*α*_*s*_ are modelled as a sum of treatment effects *γ*_*jt*_ over time and independent study effects *α*_*s*_∼Normal(0,10). Since our main interest is to investigate the performance of the models in describing the temporal patterns of the main outcomes, we will assume the variances $ \tau _{s}^{2}$ of the study outcomes as constant over time and across treatment arms. (This assumption may not hold in practice; see the real data applications below.) The main outcomes of each study, treatment and time point are reported as the sample means of the respective $\left (Z_{{isjt}}\right)_{i=1,\ldots,n_{s}}$, i.e. $Y_{{sjt}} = \left ({\sum \nolimits }_{i=1}^{n_{s}} Z_{{isjt}}\right)/n_{s}$ or

$$\begin{array}{*{20}l} Y_{{sjt}} &\sim \text{Normal }\left(\gamma_{{jt}}+\alpha_{s},\tau^{2}_{s}/n_{s}\right). \end{array} $$

Binary outcomes are simulated by taking the inverse logit transformation of *θ*_*sjt*_ and then generating individual level outcomes from a Bernoulli distribution (see (3)).

**Fig. 1:**
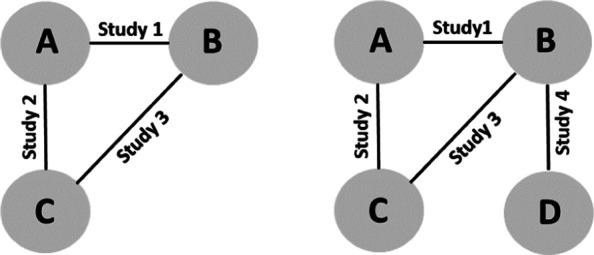
Network of studies used in the simulation scenarios. Treatments are represented as nodes and connected by an edge if a direct comparison study between them is available. The left network is closed, with one study comparing all possible pairs of treatments, while in the right network it is extended to become non-closed, containing treatment pairs not directly compared by at least one study

Different scenarios are created through various specifications of the treatment effect curves *γ*_*jt*_. Here we consider a closed network and simulate data from three hypothetical studies with two treatment arms each. See Fig. 1 (left panel) for a graphical representation. Study 1 directly compares treatments A and B with follow-up at four time points (weeks 4,8,12 and 24). Study 2 compares treatments A and C at three time points (weeks 4,12,24). Finally, study 3 compares treatments B and C at three time points (weeks 4,8,12). The number of subjects per treatment arm in the different studies are chosen as *n*_1_=100,*n*_2_=120 and *n*_3_=130. Variances are set $\tau _{1}^{2} = 1, \tau _{2}^{2} = 2$ and $\tau _{3}^{2} = 4$.

**Fig. 2:**
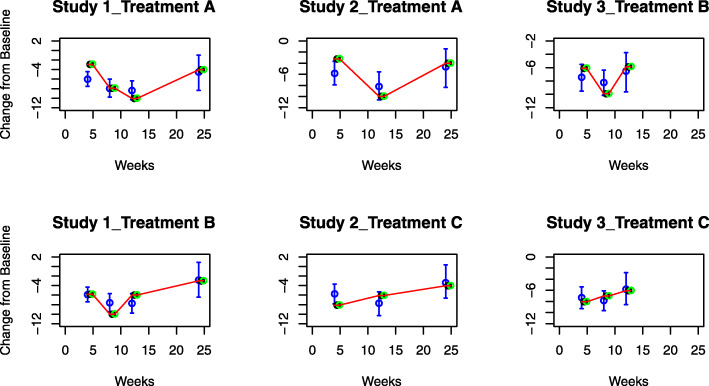
Posterior estimated profiles of *θ*_*sjt*_ obtained under the non-monotonic scenario (11). Red lines indicate the values used to simulate the data. Estimates and 95% credible intervals obtained from the respective models are indicated in blue (FPs), black (B-splines) and green (P-splines). Due to their small size, credible intervals are not visible for B-splines and P-splines

**Fig. 3:**
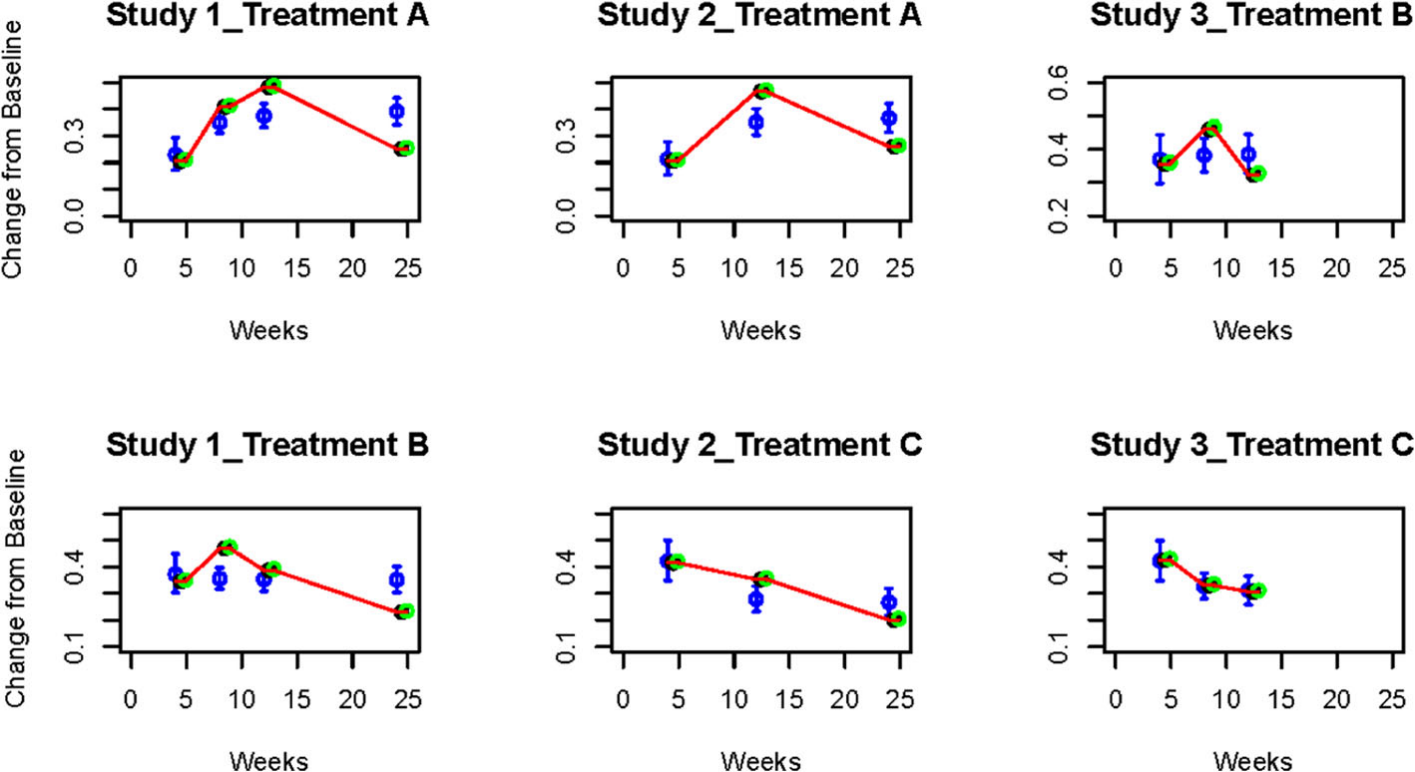
Posterior estimated profiles of *p*_*sjt*_ for binary outcomes under the non-monotonic scenario described in (11). Red lines indicate the values used to simulate the data. Estimates and 95% credible intervals obtained from the respective models are indicated in blue (FPs), black (B-splines) and green (P-splines). Due to their small size, credible intervals are not visible for B-splines and P-splines

In Figs. 2 and 3 we present, for continuous and binary outcomes respectively, a comparison of the estimated profiles obtained using the B-spline model, the P-spline model and the FP model, along with the true values used to simulate the data, assuming as treatment effects over time the non-monotonic continuous piecewise linear curves

11 $$ \begin{aligned} \gamma_{{At}}&= \left\{\begin{array}{cccc} -\frac{3t}{4}, &t\in[0,4] \\ -\frac{5t}{2}+2,&t\in(4,8] \\ -\frac{t}{2}-4, &t\in(8,12] \\ \frac{t}{2}-16, &t\in(12,24] \end{array}\right., \quad \gamma_{{Bt}}= \left\{\begin{array}{cccc} -\frac{3t}{2}, &t\in[0,4] \\ -t-2, &t\in(4,8] \\ t - 18, &t\in(8,12] \\ \frac{t}{4}-9, &t\in(12,24] \end{array}\right., \\ \gamma_{{Ct}}&= \left\{\begin{array}{cccc} -2t, &t\in[0,4] \\ \frac{t}{4}-9, &t\in(4,12]. \\ \frac{t}{6}-8, &t\in(12,24] \end{array}\right. \end{aligned}  $$

**Fig. 4:**
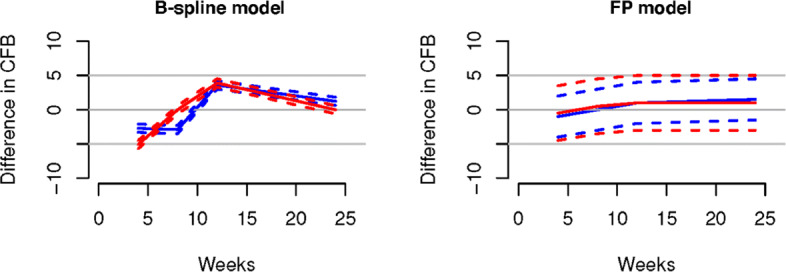
Difference in treatments for the non-monotonic simulation scenario. Treatments for A versus B (blue), and treatments A versus C (red) are shown in the left panel for the B-spline model and in the right panel for the FP model

From the Figures it is evident that the spline models are able to accurately estimate non-monotonic time effects in both the continuous and binary cases, which the FP model fails to capture. The 95% credible intervals obtained with the splines are narrow and always cover the true value used to generate the data. In Fig. 4, we show estimated differences in treatment effects for one of the 50 simulation replicates in the continuous case. It is evident that the spline models are able to capture the non-monotonic curve shape for difference in treatment, while the FP model flattens out the difference in treatment with consequent increase of uncertainty. These conclusions are confirmed by the extensive simulation study presented in Additional file 1 for other temporal patterns. We report for an overall quantitative comparison of goodness of fit the mean squared error (MSE) evaluated for each simulation scenario across all replicates. The MSE is defined as

$$\begin{array}{*{20}l} \text{MSE} = \frac{1}{R}\sum\limits_{r=1}^{R} \sum\limits_{s,j,t} \left (Y_{{sjt}}-\hat{Y}^{(r)}_{{sjt}} \right)^{2} \end{array} $$

where $\hat {Y}^{(r)}_{{sjt}}$ denotes the predicted value of the response for study *s*, treatment *j*, time *t* and MCMC iteration *r*. The number of saved MCMC iterations is *R*=10000. For all simulation scenarios the MSEs for the B-spline, P-spline and FP model are summarized in Table 2. The table reports the means (and standard deviations) of the MSEs across the 50 simulations, quantitatively showing an increase in estimation accuracy for the spline models as compared to the FP model in all scenarios, and particularly in the non-monotonic scenario simulated in (11). Further, in Table 3 we report summary statistics of the posterior estimates of *σ*_*sjt*_ obtained with the B-spline and FP model. The table confirms that the B-spline model performs better in terms of model fit and precision of the estimates. The estimates are in line with the values used to generate the data.

**Table 2:** Mean Square Error (MSE) comparison for B-spline, P-spline and fractional polynomial (FP) network meta-analysis models. Displayed are the mean and standard deviation (SD) of the respective MSEs averaged over 50 simulated data sets for each scenario. Unless stated otherwise, considered outcomes are continuous. Scenarios that contain non-monotonic temporal behaviors appear to be challenging for the FP method

Scenario	B-spline model		P-spline model		FP model	
	Mean	SD	Mean	SD	Mean	SD
Linear	0.0019	0.0213	0.0021	0.0243	0.3505	0.0388
Logarithmic	0.0014	0.0111	0.0015	0.0135	0.3505	0.0118
Piecewise linear monotonic	0.0011	0.0923	0.0052	0.1437	1.3069	0.1823
Mixed	0.0037	0.0313	0.0040	0.0931	1.5747	0.1338
Non-monotonic	0.0019	0.0389	0.0027	0.1039	41.7533	1.8354
MTC	0.0374	0.0483	0.0402	0.0984	0.9198	0.2864
BEST-ITC	0.0397	0.0503	0.0402	0.1003	0.1701	0.2898
Piecewise linear monotonic (binary)	0.0034	0.0118	0.0053	0.0978	0.0897	0.3698
Non-monotonic (binary)	0.0004	0.0019	0.0004	0.0178	0.9748	0.2976
Piecewise linear (non-closed network)	0.0172	0.0173	0.0235	0.0774	0.0903	0.3854

**Table 3:** Comparison between estimates of *σ*_*sjt*_ (*p*_*sjt*_ for binary outcomes) obtained with the B-spline, P-spline and Fractional Polynomial (FP) network meta-analysis models. We report median, minimum and maximum evaluated over the 50 replicates of the average *σ*_*sjt*_ (respectively *p*_*sjt*_) for each scenario

Scenario	B-spline model			FP model		
	Median	Min	Max	Median	Min	Max
Linear	0.1007	0.1003	0.1010	0.1423	0.1172	0.1867
Logarithmic	0.1016	0.1014	0.1018	0.1439	0.1258	0.1761
Piecewise linear monotonic	0.1300	0.1190	0.1468	0.5701	0.5552	0.5875
Mixed	0.1321	0.1200	0.1497	0.6794	0.6666	0.6850
Non-monotonic	0.1399	0.1240	0.1760	0.9321	0.9321	0.9322
MTC	0.0829	0.0746	0.0939	0.1831	0.1236	0.3685
BEST-ITP	0.1352	0.1262	0.1426	0.2507	0.1712	0.4110
Piecewise linear monotonic (binary)	0.6128	0.5923	0.6246	0.6989	0.6830	0.7037
Non-monotonic (binary)	0.5940	0.5763	0.6011	0.7732	0.7372	0.8189
Piecewise linear (non-closed network)	0.1483	0.1203	0.1749	1.0382	1.0365	1.0408

### Real data application

**Fig. 5:**
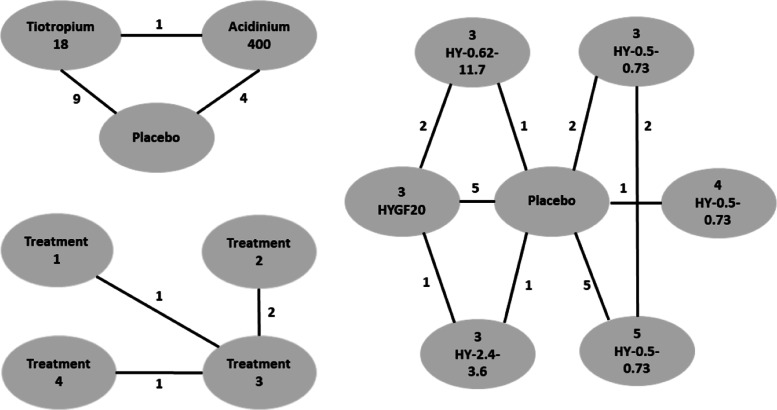
Networks of studies of the COPD data set (top left), the T2D data set (bottom left) and the OA data set (right). Edges indicate the number of studies providing direct comparison between adjacent treatments

**Fig. 6:**
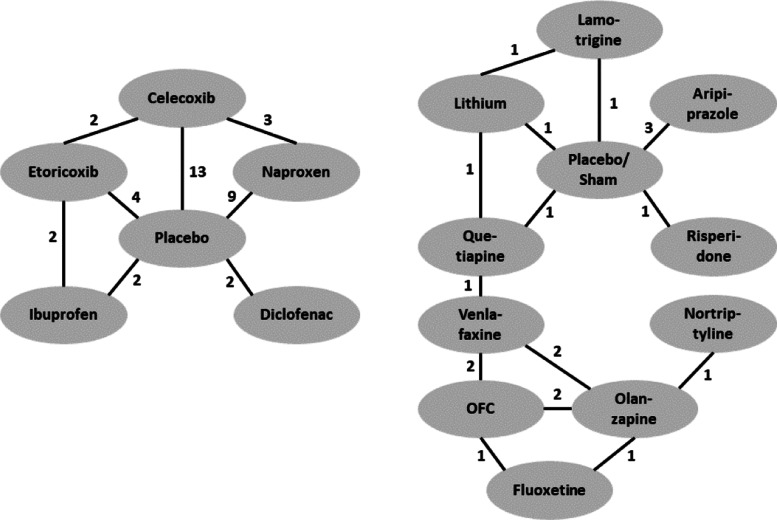
Networks of studies of the CMP data set (left) and the MDD data set (right). Edges indicate the number of studies providing direct comparison between adjacent treatments

We consider five real NMA data sets. The networks of studies for all data sets are provided in Figs. 5 and 6. Main features of all data sets are summarized in Table 4.

- The first data set contains information from 13 studies on patients affected by chronic obstructive pulmonary disease (COPD) and is described in [35] and [7]. COPD is a long term lung condition affecting adults worldwide, for which there is no cure but treatment can help slow down disease progression. Subjects receive one of three possible treatments: Aclidinium 400*μ*g BID (AB400), Tiotropium 18*μ*g QD (TIO18), or placebo. Three studies compare AB400 with placebo, nine compare TIO18 with placebo, and one compares AB400 and TIO18 with placebo. There is one closed loop, providing direct evidence. As all studies are placebo-controlled, placebo is used as the reference treatment.

**Table 4:** Summary of main features of the chronic obstructive pulmonary disease (COPD), osteroarthritis (OA), type 2 diabetes (T2D), chronic muskuloskeletal pain (CMP), and treatment resistant major depressive disorder (MDD) data sets

	COPD	OA	T2D	CMP	MDD
Number of studies	13	16	4	27	13
of treatments	3	7	4	6	10
Number of follow ups	2–6	1–10	2–5	2	3–4
Number of patients per study	46–3006	20–295	88–575	12–6769	24–1147
Network type	closed	non-closed	non-closed	non-closed	non-closed
- The second data set contains information from 16 studies on treatments for osteoarthritis (OA) of the knee and is described in [10] and [7]. OA is a painful chronic degenerative joint condition. The treatments in the systematic review are based on different hyaluronan (HA)-based viscosupplements. The different treatments considered are: three, four, or five injections of HA with a molecular weight (MW) of 0.5-0.73 million Da (Hyalgan) (3 Hy-0.5-0.73; 4 Hy-0.5-0.73; 5 Hy-0.5-0.73); three injections of HA MW of 0.62-11.7 million Da (Supartz) (3 Hy-0.62-11.7); three injections of HA MW of 2.4-3.6 million Da (Euflexxa) (3 Hy-2.4-3.6); and three injections of Hylan GF-20 MW 6 million Da (Synvisc) (3 HyGF20). Placebo is the baseline treatment.
- The third data set contains information from 4 studies on patients affected by type 2 diabetes (T2D) and is described in [9] and [[7], Supplementary Materials]. Diabetes poses an enormous individual and societal burden, with high risk of major complication and diminished quality and length of life. The authors consider four treatments of which one is placebo and the others are oral anti-diabetic agents. Since the authors do not give further details we also label the treatments as 1 through 4, the baseline being the placebo treatment 1. The primary clinical outcome of interest is the hemoglobin A1c (HbA1c, %) reduction from baseline, with larger absolute value indicating better efficacy.
- The fourth data set is part of a large collection of studies on benefits and risks of drugs for treating chronic musculoskeletal pain (CMP) in patients with osteoarthritis or rheumatoid arthritis. CMP disorders are associated with some of the poorest health related quality of life. Patients with pain experience severe restrictions on their functioning and ordinary daily activities. The data are collected in [36] and the authors conduct a Bayesian NMA. Here we concentrate on pain relief as the outcome of interest, measured by visual analogue scale (VAS), and only include studies with two follow-ups. Treatments are Diclofenac, Naproxen, Ibuprofen, Celecoxib, and Etoricoxib, which are compared to placebo. Outcomes are reported at 6 and 12 weeks (within 2-week range).
- The fifth data set is taken from a comparative review of efficacy and tolerability of interventions for treatment resistant major depressive disorder (MDD) [37]. MDD is a mental disorder affecting about 10−15*%* of the general population. It is associated with depressed mood and/or loss of interest or pleasure in daily activities for more than two weeks. MDD is associated with significant morbidity and mortality, and often patients fail to achieve full remission. Here we focus on efficacy outcome measured as CFB in the Montgomery-Asberg Depression Rating Scale (MADRS). Interventions are Aripiprazole, Fluoxetine, Lamotrigine, Lithium, Nortriptyline, Olanzapine, Olanzapine/Fluoxetine combination (OFC), Quetiapine, Risperidone, Venlafaxine, which are compared to placebo/sham. Outcomes are reported at 4,6 or 8 weeks.

For the data sets for which no information about the loss to follow-up is available, we assume that the sample sizes remain the same as at the start of the study. For studies within the NMAs that do not report the number of patients, we generate the sample size from a uniform distribution ranging from the minimum to the maximum number of patients across all studies of the respective NMA. Finally, following [8], we impute any missing standard deviation information specifying the prior distributions

$$\begin{array}{*{20}l}\text{sd}_{{sjt}} &\sim \text{Gamma} \left(\alpha_{1}, \alpha_{2}\right),\\ \alpha_{i} &\sim \text{Uniform} (0,10). \end{array} $$

**Fig. 7:**
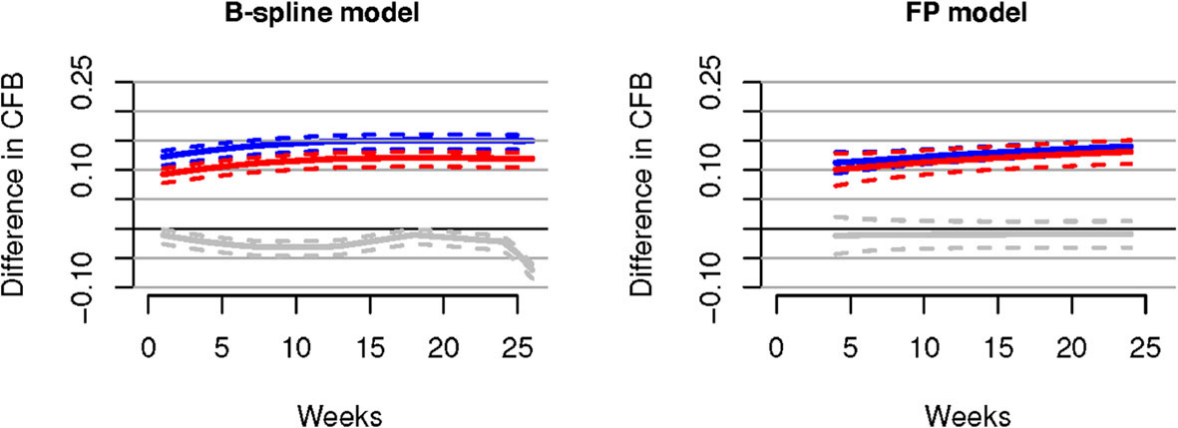
Results for the chronic obstructive pulmonary disease (COPD) data set. Shown are estimation results as mean differences in treatment effects over time for AB400 versus placebo (red), TIO18 versus placebo (blue) and AB400 versus TIO18 (grey) generated by the B-spline model (left panel) and the FP model (right panel). Dashed lines denote 95% credible intervals, while solid lines represent the posterior mean. These results are in agreement with the conclusions in [35]

**Fig. 8:**
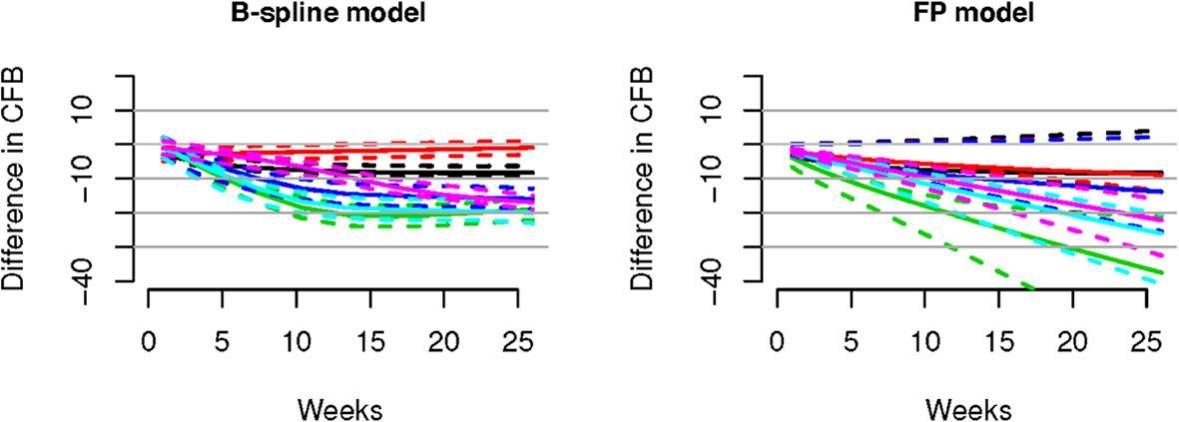
Results for the osteoarthritis (OA) data set. Shown are treatment effects relative to placebo over time for 5 HY-0.5-0.73 (magenta), 4 HY-0.5-0.73 (black), 3 HY-0.5-0.73 (red), 3 HYGF20 (green), 3 HY-0.62-11.7 (blue) and 3 HY-2.4-3.6 (light blue) estimated by the B-spline model (left panel) and the FP model (right panel). Dashed lines denote 95% credible intervals, while solid lines represent the posterior means. Both models suggest that the best results are obtained by 3 HYGF20, in agreement with the result obtained originally for this data with FPs by [10]

**Fig. 9:**
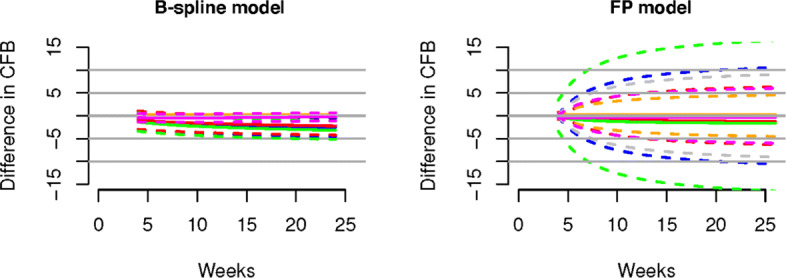
Results for the type 2 diabetes (T2D) data set. Shown are mean differences in treatment effects over time for treatments 1 versus 2 (blue), 1 versus 3 (red), 1 versus 4 (green), 2 versus 3 (orange), 2 versus 4 (grey) and 3 versus 4 (magenta) generated by the B-spline model (left panel) and FP model (right panel). Dashed lines denote 95% credible intervals, while solid lines represent posterior means

**Fig. 10:**
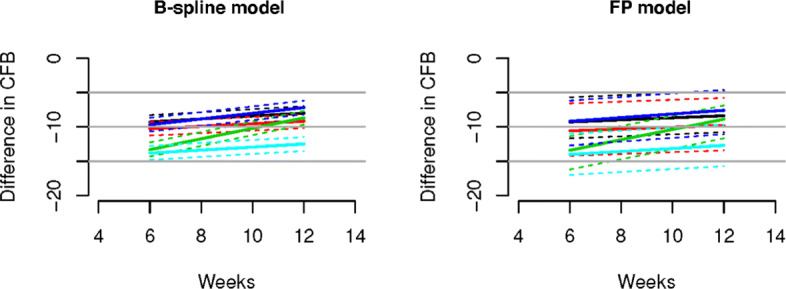
Results for chronic musculoskeletal pain (CMP) data set. Shown are treatment effects relative to placebo over time for Celecoxib (black), Naproxen (red), Etoricoxib (green), Ibuprofen (blue), and Diclofenac (light-blue) estimated using the B-Spline model (left panel) and the FP model (right panel). Dashed lines denote 95% credible intervals, while solid lines represent posterior means. Both figures are in agreement with the result of [36] that Diclofenac is the most effective

**Fig. 11:**
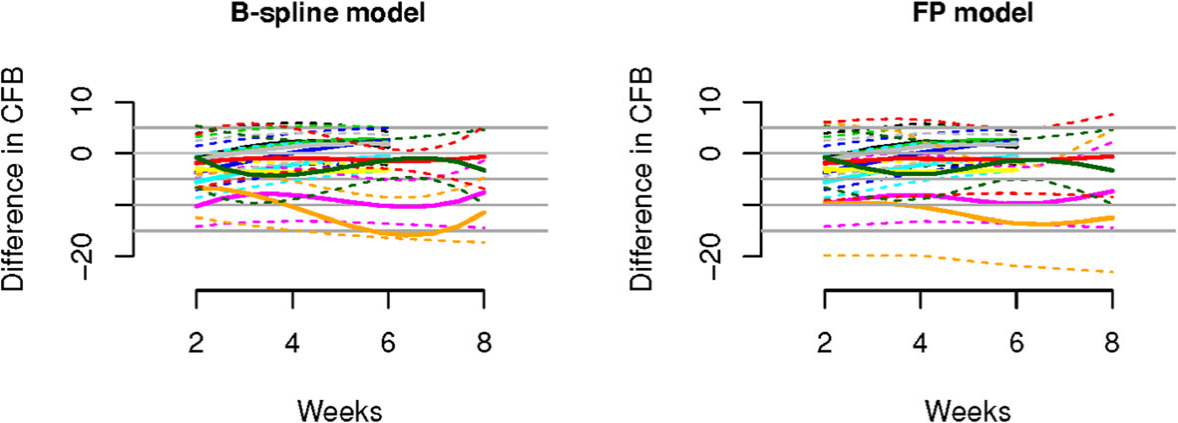
Results for the major depressive disorder (MDD) data set. Shown are treatment effects relative to placebo over time for Nortriptyline (black), Lithium (red), Fluoxetine (green), Olanzapine (blue), OFC (ligth-blue), Risperidone (magenta), Aripiprazole (yellow), Venlafaxine (grey), Quetiapine (orange) and Lamotrigine (dark green) estimated using the B-Spline model (left panel) and the FP model. Dashed lines denote 95% credible intervals, while solid lines represent posterior means. The results are in qualitative agreement with [37]

Posterior inference results are shown in Figs. 7, 8, 9, 10, 11.

## Discussion

From the simulation study (see Figs. 2, 3 and Table 2) (see Additional file 1 for further scenarios) it appears evident that the results obtained from the B-spline and the P-spline models are virtually identical. This is not surprising due to the use of the same basis functions and the small number of time points and knots in our application, resulting in a very small number of second order differences. Hence the local smoothing, the most attractive feature of P-splines, has very limited opportunity. Moreover, penalization is naturally incorporated in the Bayesian framework through the prior distribution. Indeed, the prior in (8) already shrinks small coefficients to zero while keeping substantial effects large. Furthermore, it is well known that the prior distribution performs a similar role as the penalty term in classical penalized regression, with the advantage that the penalty parameters can be estimated jointly with the other parameters of the model. From a Bayesian perspective, the main difference between the priors in (8) and in (9) is that the former assumes globally constant smoothing while the latter facilitates locally adaptive smoothing. This is relevant in applications with larger time horizon and appealing when the underlying function is oscillating. Given the limited number of time observations and the often regular behaviour of treatment effects over time in our application, the difference between B-splines and P-splines is negligible. For this reason we only fit the B-spline model in the real data applications.

The simulation study results show that the spline based models offer a flexible strategy that is able to accommodate different time patterns and non-linearities in the underlying curve without the computational burden of the FP model, while still leading to better results. Cubic splines with four uniformly spaced knots appear to be able to provide accurate estimates and seem a natural choice, since it is common in NMA that few time measurements are available per treatment arm and study.

A possible extension of our model is the inclusion of within-study correlation between different time points. It has been shown that ignoring it might lead to increased residual variability, standard error of the pooled estimates and mean squared error. Including a specific parameter for within-study variability influences the borrowing of strength between time points and ultimately across studies. These considerations have been discussed in the literature and different methods to accommodate within-study correlation in meta-analysis have been proposed [see, e.g. [38, 39]]. In our work, the within-study correlation is mostly accounted for by flexibly modelling the mean function, which describes the time evolution of the treatment effect, by using B-splines. This strategy allows to capture structure in the data. Conditionally on the mean function and the remaining parameters in the model, the observations are independent. Borrowing of strength for inference across studies is mainly achieved through the joint modelling of the study-specific mean functions. In this framework it is in principle possible to include correlated errors among observations at different time points, and some proposals in this direction can be found in the literature. Here we do not introduce this further source of uncertainty given the limited amount of time-points per study per treatment, the fact that the observations are rarely close in time, the use of aggregated data, and finally because often (as shown in our data examples) the time pattern of the treatment effect is not noticeably oscillating but, on the contrary, is often linear or piecewise linear. Moreover, in the case of continuous responses we rescale in (4) the variances of the main outcomes, which achieves a similar effect as the rescaling of the variance by the autoregressive coefficient in an Autoregressive Model of order 1. For different types of responses other rescaling strategies need to be adopted. For example, for binomial or count data explicit correlation between time points could be introduced by modelling the outcome variables using a multivariate Binomial or a multivariate Poisson distribution, respectively.

The results of the real data applications confirm those of the simulation study. For the FP fit we considered first and second order FPs. The estimated profiles using the B-spline and FP models are presented in Figs. 7-9. B-splines provide narrower credible intervals for the posterior mean estimates, while still being able to detect slight changes over time. As a rule of thumb, if the 95% credible interval of the posterior distribution of the difference between two treatments does not cover zero, then we can conclude there is evidence in support of different effectiveness between them. In the results for the OA data it is evident that the B-spline model is able to detect non-monotonic time patterns, while the FP model forces a monotonic trend given the parameter choice which is dictated by the DIC. Furthermore, for the T2D and the CMP examples, the shape and size of the credible intervals obtained with the FP model is due to the choice (by DIC) of a first order polynomial with power *p*=−2. Any choice of FP parameters generally imposes structure on the temporal pattern, which might not always be supported by the data. In particular, this is the case for the choice of the function *β*/*t*^2^ when, as for the CMP data, only two data points per study and treatment arm are available. In Table 5 we report the specific FP selected by DIC for each data set. These examples confirm that the DIC might not be the optimal model choice criterion when confronted with non-linear effects. Indeed, while the DIC has been shown to be an approximation to a penalized loss function based on the deviance with a penalty derived from a cross-validation argument, it has been warned that this approximation is in fact only valid when the effective number of parameters *p*_eff_ in the model is considerably smaller than the number of independent observations *n* [see [20]]. Since the latter assumption is usually not satisfied in the case of NMAs the DIC can tend to under-penalize more complex models. The poor empirical performance of DIC compared to cross-validation when the assumption *p*_eff_≪*n* is violated has also been highlighted by [21].

**Table 5:** Real data sets: DIC selection of the order *M*_*F*_ and of the power terms *p*_*m*_ in the FP model. RE and FE refer to random and fixed effects

Data set	*M*_*F*_	*p*_1_	*p*_2_	RE/FE
COPD	2	-0.5	0	RE on *β*_2_
OA	2	0.5	1	RE on *β*_2_
T2D	1	-2		RE on *β*_1_
CMP	1	-1		FE
MDD	2	0.5	0	FE

## Conclusions

In this article we propose a random effect model for NMA of repeated measurements based on splines. The model is able to accommodate a large class of temporal treatment effect patterns, allowing for direct and indirect comparisons of widely varying shapes of longitudinal profiles. We argue for a fixed choice of the order and automatic selection of uniformly spaced knots of the B-spline. The model is not restricted to continuous or binary outcomes, but can be extended to any type of response variable by specifying an appropriate link function. An important extension of the model is to account for the correlation structure between trial specific treatment effects, $\delta _{sj_{i}}$ and $\delta _{sj_{k}}$, in multiple treatment arm studies. This can be achieved by decomposing the multivariate Normal distribution into a sequence of conditional univariate distributions as described in [10, 26]. In the context of longitudinal NMA, B-spline and P-spline models provide equivalent posterior inference but we believe that B-splines achieve a satisfactory level of penalization and smoothness for this application. Furthermore, we show that the B-spline model overcomes the methodological and computational limitations of the FP approach, which, beyond requiring extensive sensitivity analysis for each new scenario, might also suffer from potential shortcomings of the DIC. In detail investigation of model choice criteria for FP selection is needed, but it is beyond the scope of this work. One of the main consequences of a sub-optimal choice of the polynomial can be observed in the uncertainty quantification as represented by the credible intervals for the FP model. The B-spline model is useful in understanding treatment effects as well as between-study variability and naturally allows for different numbers of observations per study, different times of observations, different sample sizes across studies, as well as missing data, and there are arguments for better interpretability of its coefficients as compared to the FP model. The concerns of [10], about splines with fixed number of uniformly spaced knots being too restrictive in possible curve shapes, are not supported by our results in simulations and real data applications, which highlight the flexibility and efficiency of the B-spline approach.

## Supplementary information

**Additional file 1** Table S6 contains simulated temporal patterns for additional simulation scenarios (i) linear, (ii) logarithmic, (iii) piecewise linear monotonic, (iv) mix of the previous with one treatment effect being constant. Figures S12, S13, S14 and S15 show respective estimated profiles obtained for the B-spline, P-spline and FP models, along with true values used to simulate the data. Figure S16 shows estimated profiles for case (iii) with binary outcomes. Figure S17 shows estimated profiles for a scenario, as in the “Simulation study” section, in which temporal patterns have been generated according to the mixed treatment comparison (MTC) model. Figure S18 shows estimated profiles for a scenario in which temporal patterns have been generated from the Bayesian evidence synthesis techniques – integrated two-component prediction (BEST-ITP) model. Figure S19 illustrates the influence of a non-closed network.

**Additional file 2** Figures S20, S21 and S22 contain MCMC traceplots.

**Additional file 3** JAGS code for B-spline model.

**Additional file 4** R code to generate simulation scenario data.

## Data Availability

Data sets for the real data examples are publicly available in the cited references.

## Abbreviations

BEST-ITP: Bayesian evidence synthesis techniques –integrated two-component prediction
CFB: change from baseline
CMP: chronic muskuoskeletal pain
COPD: chronic obstructive pulmonary disease
DIC: deviance information criterion
FP: fractional polynomial
MCMC: Markov Chain Monte Carlo
MDD: major depressive disorder
MSE: mean squared error
MTC: mixed treatment comparison
NMA: network meta-analysis
T2D: type 2 diabetis
