# Supplementary material for: Bayesian splines versus fractional polynomials in network meta-analysis
Source: BMC Med Res Methodol. 2020 Oct 20;20:261. doi: 10.1186/s12874-020-01113-9 (PMC7574305; doi:10.1186/s12874-020-01113-9)
Supplement: Supplementary file 1 — Additional file 1 Table S6 contains simulated temporal patterns for additional simulation scenarios (i) linear, (ii) logarithmic, (iii) piecewise linear monotonic, (iv) mix of the previous with one treatment effect being constant. Figures S12, S13, S14 and S15 show respective estimated profiles obtained for the B-spline, P-spline and FP models, along with true values used to simulate the data. Figure S16 shows estimated profiles for case (iii) with binary outcomes. Figure S17 shows estimated profiles for a scenario, as in the “Simulation study” section, in which temporal patterns have been generated according to the mixed treatment comparison (MTC) model. Figure S18 shows estimated profiles for a scenario in which temporal patterns have been generated from the Bayesian evidence synthesis techniques – integrated two-component prediction (BEST-ITP) model. Figure S19 illustrates the influence of a non-closed network. [file 12874_2020_1113_MOESM1_ESM.pdf]

Table and Figures for Additional file 1

Additional file 1: Table S6 contains simulated temporal patterns for additional simulation scenarios (i) linear, (ii) logarithmic, (iii) piecewise linear monotonic, (iv) mix of the previous with one treatment effect being constant. Figures S12, S13, S14 and S15 show respective estimated profiles obtained for the B-spline, P-spline and FP models, along with true values used to simulate the data. Figure S16 shows estimated profiles for case (iii) with binary outcomes. Figure S17 shows estimated profiles for a scenario, as in the Simulation Study Section, in which temporal patterns have been generated according to the mixed treatment comparison (MTC) model. Figure S18 shows estimated profiles for a scenario in which temporal patterns have been generated from the Bayesian evidence synthesis techniques – integrated two-component prediction (BEST-ITP) model. Figure S19 illustrates the influence of a non-closed network.

Table S6 Temporal patterns for treatment effects of treatments A,B and C used in the simulations shown in Figures S12-S15. The mixed scenario contains a constant treatment effect for treatment B and a quadratic effect for treatment C. The simulated temporal patterns γ_{At}, γ_{Bt} and γ_{Ct} in the scenarios are (i) linear, (ii) logarithmic, (iii) piecewise linear monotonic, and (iv) a mix of the above with one of the treatment effects being constant.

Scenario	γ_{At}	γ_{Bt}	γ_{Ct}
(i)	$-t/4$	$-t/2$	$-t/3$
(ii)	$-\log(t+1)$	$-3\log(t+1)$	$-2\log(t+1)$
(iii)	$\begin{cases} -\frac{3t}{4}, & t \in [0,4] \\ -\frac{t}{2}-1, & t \in (4,8] \\ -\frac{t}{4}-3, & t \in (8,12] \\ -6, & t \in (12,24] \end{cases}$	$\begin{cases} -\frac{3t}{2}, & t \in [0,4] \\ -\frac{10t}{23}-\frac{17}{4}, & t \in (4,8] \\ -\frac{5t}{16}-\frac{21}{4}, & t \in (8,12] \\ -\frac{t}{12}-8, & t \in (12,24] \end{cases}$	$\begin{cases} -t, & t \in [0,4] \\ -\frac{3t}{4}-1, & t \in (4,8] \\ -\frac{t}{4}-5, & t \in (8,12] \\ -\frac{t}{12}-7, & t \in (12,24] \end{cases}$
(iv)	$\begin{cases} 0, & t \in [0,8] \\ -\frac{t}{2}+4, & t \in (8,24] \end{cases}$	0	$(-t^2-25t)/100$

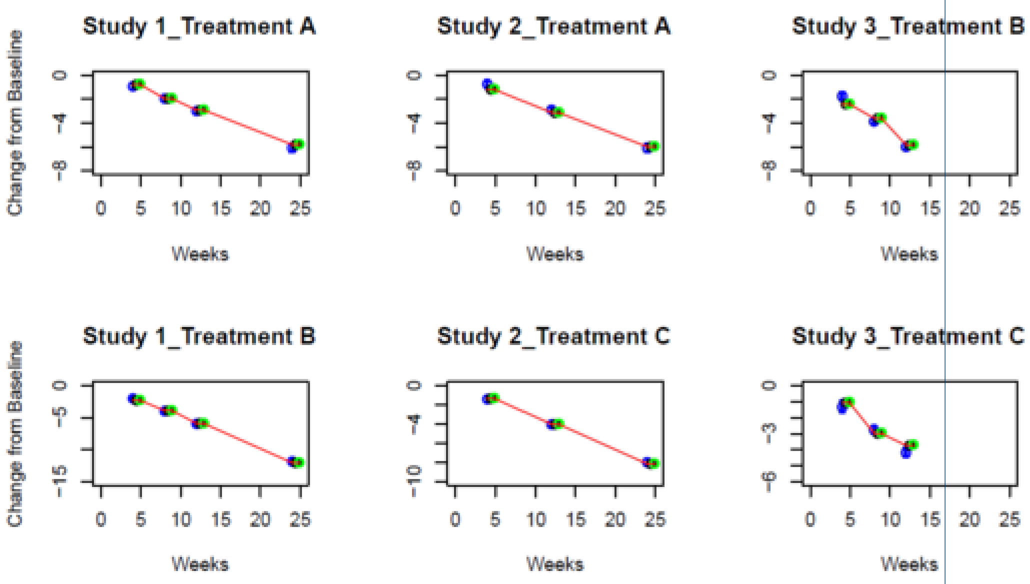

Figure S12 Posterior estimated profiles obtained for the linear scenario. Red lines indicate the values used to simulate the data. Estimates and 95% credible intervals obtained from the respective models are indicated in blue (FPs), black (B-splines) and green (P-splines). Due to their small size, credible intervals are not visible.

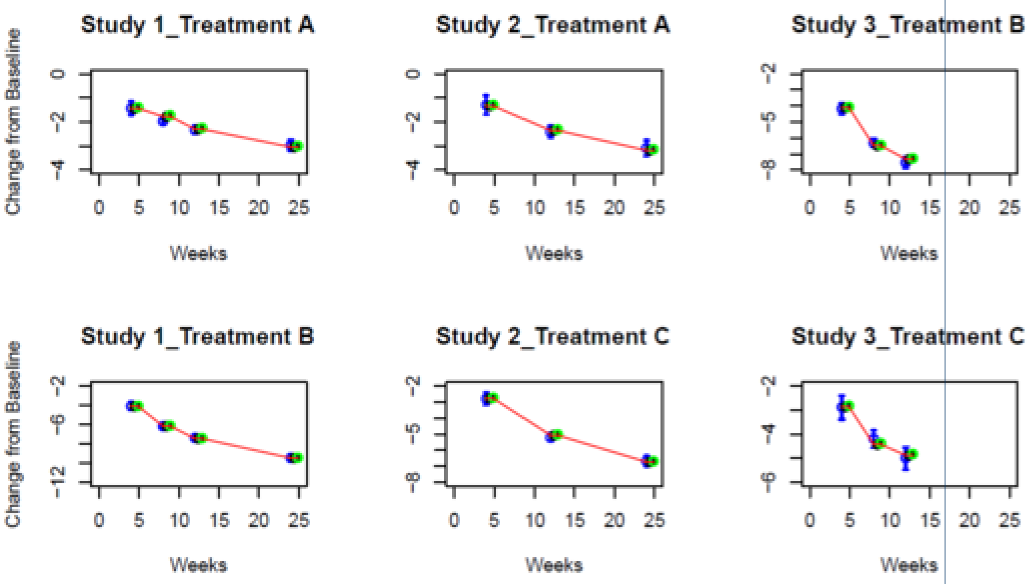

Figure S13 Posterior estimated profiles obtained for the logarithmic scenario. Red lines indicate the values used to simulate the data. Estimates and 95% credible intervals obtained from the respective models are indicated in blue (FPs), black (B-splines) and green (P-splines). Due to their small size, credible intervals are not visible for B-splines and P-splines.

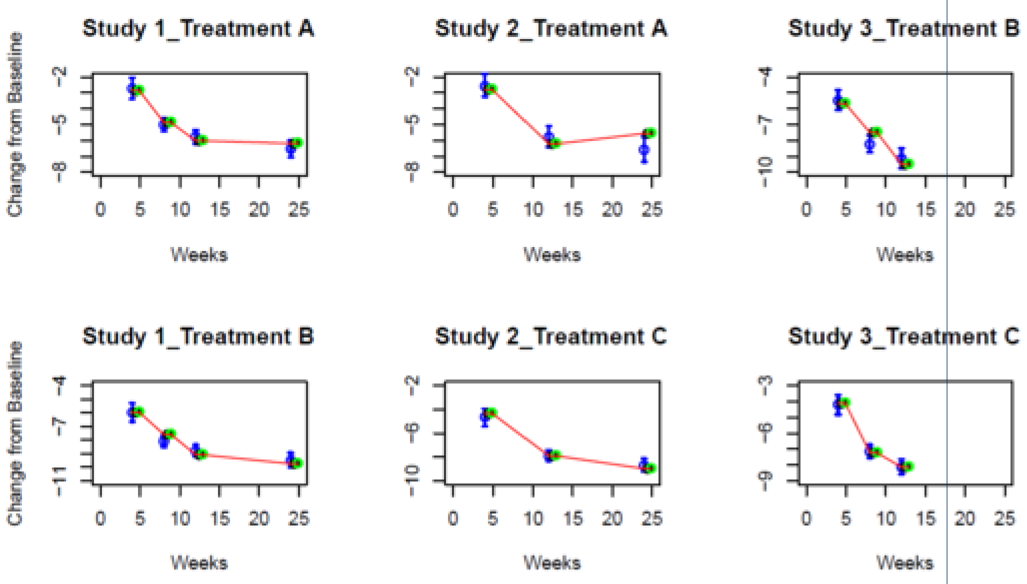

Figure S14 Posterior estimated profiles obtained for the piecewise linear monotonic scenario. Red lines indicate the values used to simulate the data. Estimates and 95% credible intervals obtained from the respective models are indicated in blue (FPs), black (B-splines) and green (P-splines). Due to their small size, credible intervals are not visible for B-splines and P-splines.

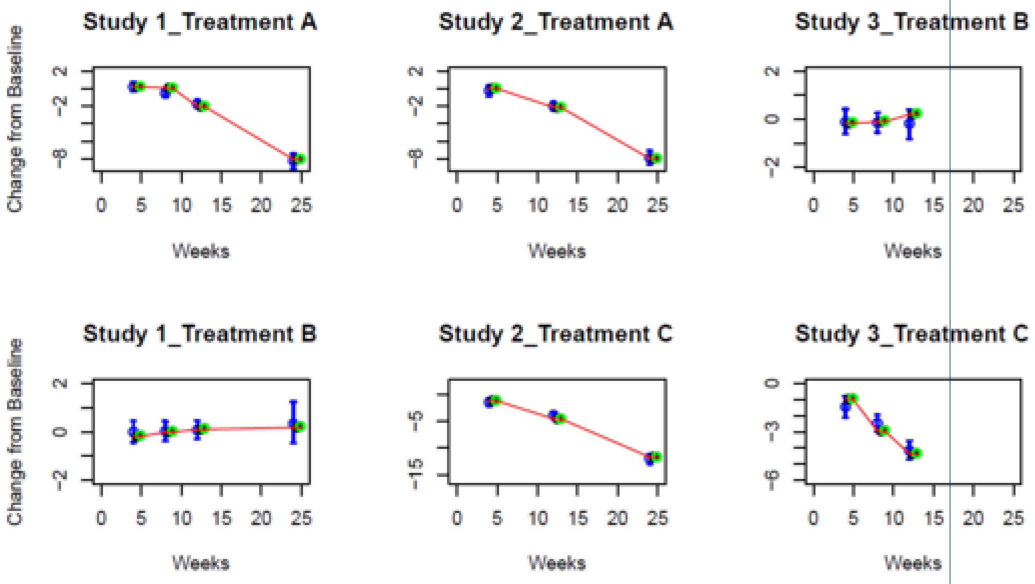

Figure S15 Posterior estimated profiles obtained for the mixed scenario. Red lines indicate the values used to simulate the data. Estimates and 95% credible intervals obtained from the respective models are indicated in blue (FPs), black (B-splines) and green (P-splines). Due to their small size, credible intervals are not visible for B-splines and P-splines.

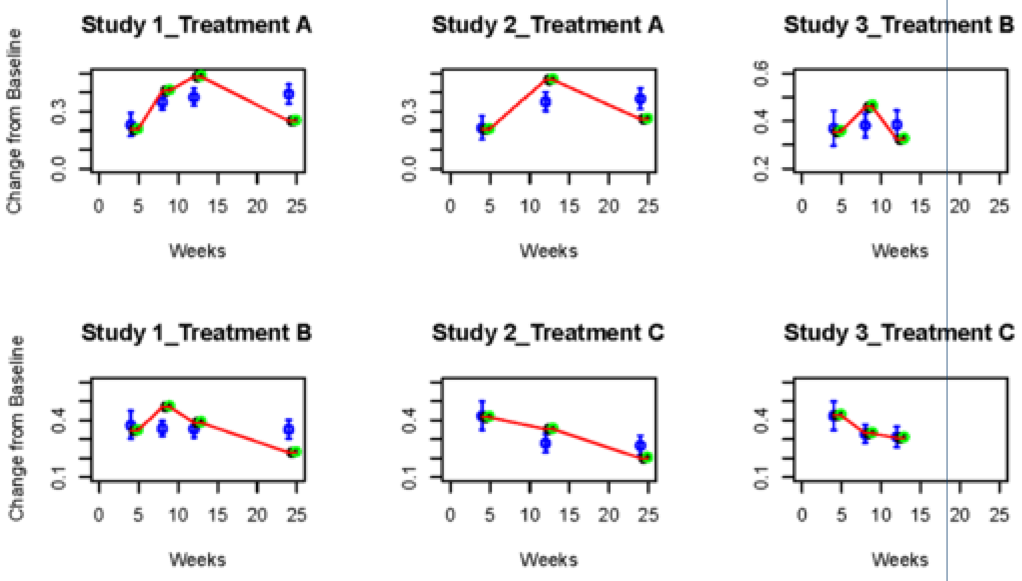

Figure S16 Posterior estimated profiles obtained for the piecewise linear scenario with binary outcomes. Red lines indicate the values used to simulate the data. Estimates and 95% credible intervals obtained from the respective models are indicated in blue (FPs), black (B-splines) and green (P-splines). Due to their small size, credible intervals are not visible for B-splines and P-splines.

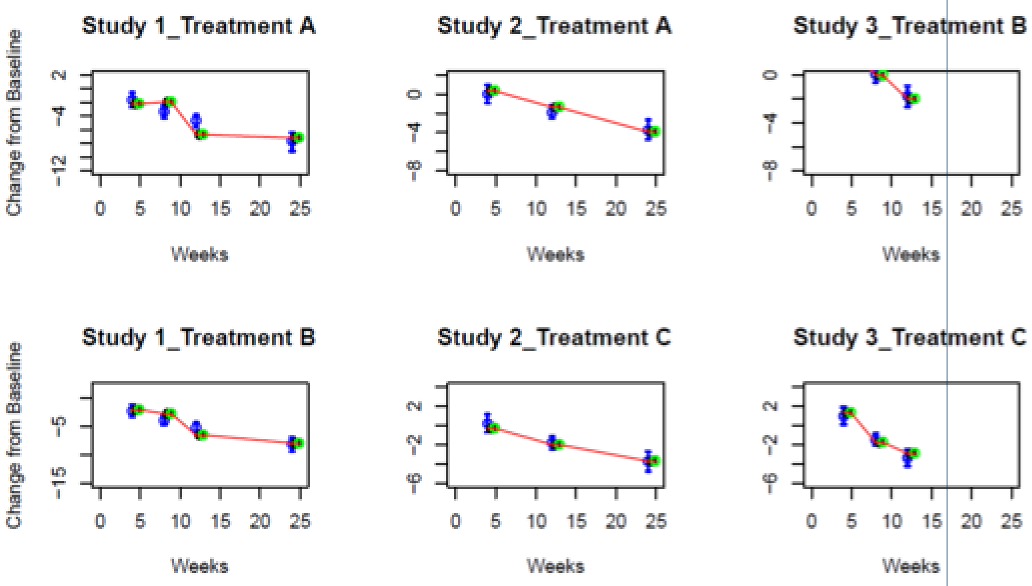

Figure S17 Posterior estimated profiles obtained for the MTC scenario. Red lines indicate the values used to simulate the data. Estimates and 95% credible intervals obtained from the respective models are indicated in blue (FPs), black (B-splines) and green (P-splines). Due to their small size, credible intervals are not visible for B-splines and P-splines. Mean outcomes are modelled as $\theta_{sjt} = \mu_{st} + \delta_{sj}$, the sum of a study- and time-specific effect across treatment arm and a study-specific arm deviation, where $\mu_{st} \sim$ Normal $(-s, 1)$ and $\delta_{sj} \sim$ Normal $(0.5, 1)$, i.e., in this model relative treatment effects are assumed to be constant over time. Simulation data is generated with $\sigma_{sjt} = 1.2/\sqrt{n_s}$, i.e., assuming no within-study correlation between subsequent time points.

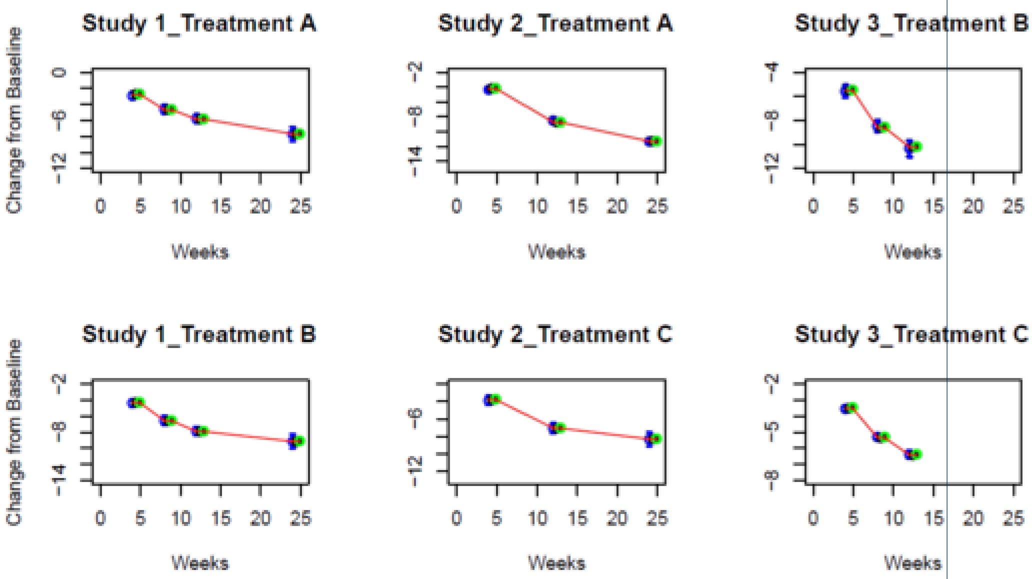

Figure S18 Posterior estimated profiles obtained for the BEST-ITP scenario. Red lines indicate the values used to simulate the data. Estimates and 95% credible intervals obtained from the respective models are indicated in blue (FPs), black (B-splines) and green (P-splines). Due to their small size, credible intervals are not visible for B-splines and P-splines. Mean outcomes are modelled as $\theta_{sjt} = (\phi_s + \delta_j)(1 - e^{-p_j t})/(1 - e^{-p_j T})$, where $\phi_s \sim$ Normal $(-3, 1)$, and $\delta_1 = 0$, $\delta_2 = 0.5$, $\delta_3 = 1$ are treatment effects at the end follow-up time T, to which they increase according the shape given by the exponential function factor with $p_1 = -0.1$, $p_2 = -0.15$ and $p_3 = -0.15$.

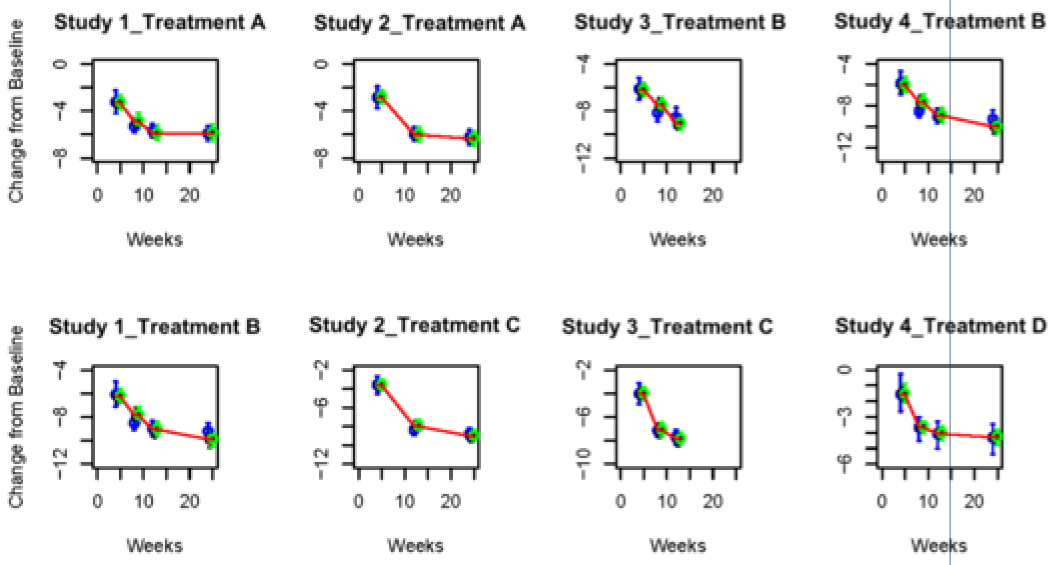

Figure S19 Posterior estimated profiles obtained for the piecewise linear monotonic scenario (iii) under the non-closed network in Figure ?? (right graph). The additional treatment D, which the extra study 4 directly compares with treatment B, is given by the temporal effect pattern $\gamma_{Dt} = -t$ for $t \in [0, 4]$, $\gamma_{Dt} = -3t/4 - 1$ for $t \in (4, 8]$, $\gamma_{Dt} = -t/4 - 5$ for $t \in (8, 12]$ and $\gamma_{Dt} = -t/12 - 7$ for $\in (12, 24]$. Follow-up times of study 4 are placed at weeks $4, 8, 12$ and 24, the number of observations is chosen as $n_4 = 110$, and the variance as $\tau_4^2 = 3$. Red lines indicate the values used to simulate the data. Estimates and 95% credible intervals obtained from the respective models are indicated in blue (FPs), black (B-splines) and green (P-splines). Due to their small size, credible intervals are not visible for B-splines and P-splines.
