# Supplementary material for: Bayesian splines versus fractional polynomials in network meta-analysis
Source: BMC Med Res Methodol. 2020 Oct 20;20:261. doi: 10.1186/s12874-020-01113-9 (PMC7574305; doi:10.1186/s12874-020-01113-9)
Supplement: Supplementary file 2 — Additional file 2 Figures S20, S21 and S22 contain MCMC traceplots. [file 12874_2020_1113_MOESM2_ESM.pdf]

Figures for Additional file 2

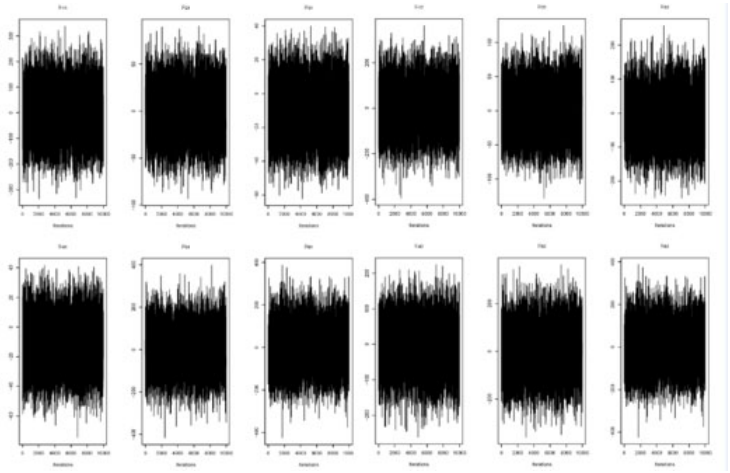

Figure S20 Non-monotonic scenario, B-spline model: Traceplots of $\boldsymbol{\mu}_1$ (left six panels) and $\boldsymbol{\mu}_2$ (right six panels).

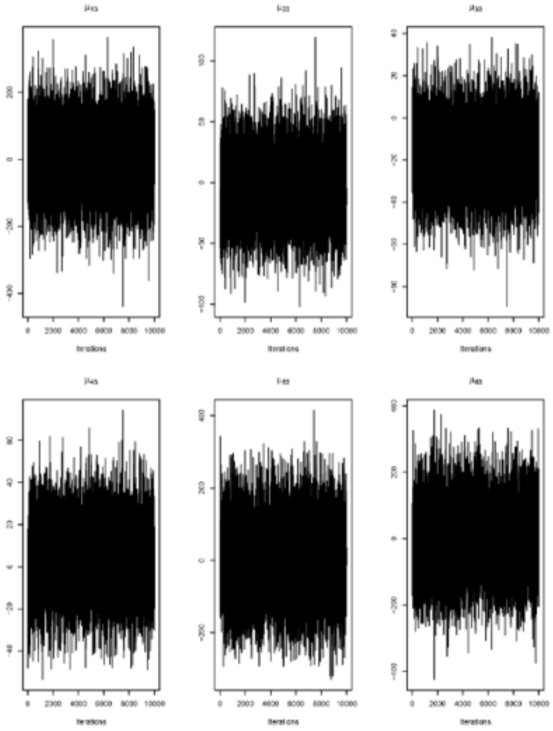

Figure S21 Non-monotonic scenario, B-spline model: Traceplots of $\boldsymbol{\mu}_3$.

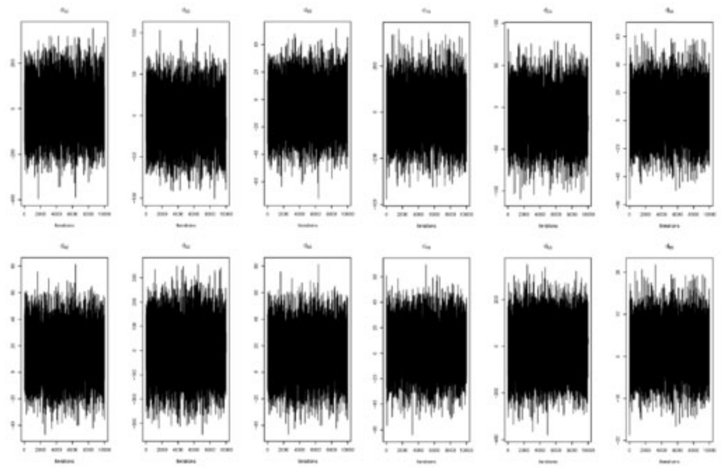

Figure S22 Non-monotonic scenario, B-spline model: Traceplots of $\boldsymbol{d}_2$ (left six panels) and $\boldsymbol{d}_3$ (right six panels).
