# Supplementary material for: Bayesian splines versus fractional polynomials in network meta-analysis
Source: BMC Med Res Methodol. 2020 Oct 20;20:261. doi: 10.1186/s12874-020-01113-9 (PMC7574305; doi:10.1186/s12874-020-01113-9)
Supplement: Supplementary file 3 — Additional file 3 JAGS code for B-spline model. [file 12874_2020_1113_MOESM3_ESM.pdf]

Additional File 3

```
# JAGS code for the B-spline model in
# "Bayesian splines versus fractional polynomials in network meta-analysis"
# by A. Heinecke, M. Tallarita, M. De Iorio

model{
  se_alpha ~ dunif(0,10)
  se_beta  ~ dunif(0,10)

  for (i in 1:nObs){  ## nObs is the total number of records
    ## model for sd of the response if sd is unknown
    se_na[i] ~ dgamma(se_alpha,se_beta)T(1,)
    se_new[i]<-(se[i]*I_se[i]+se_na[i]*(1-I_se[i]))/sqrt(n[i])
    ## I_se is binary, indicating whether sd is known or unknown
    invsigma[i]<-(1-corr*corr)/(se_new[i]*se_new[i])  ## variance of response

    y[i] ~ dnorm(theta[i], invsigma[i]) ## model for the response

    theta[i]<- sum(Beta[s[i],a[i],]*x[time[i],])
    ## x represents the basis expansion, a indicates treatment
  }

  for (l in 1:Ns){ ## Ns is the total number of studies
    for (ll in 1:na[l]){ ## na indicates the treatment for study l
      for (lll in 1:Nbasis){ ## Nbasis is total number of basis functions
        Beta[l,ll,lll]<-mu[l,lll]+d[t[l,ll],lll]-d[t[l,1],lll]
      }
    }
  }

  ## Prior Specification
  for (j in 1:Ns){
    mu[j,1:Nbasis] ~ dmnorm(mean_mu[],prec_mu[,])
  }
  d[1,1:Nbasis]<-0  ## force d[1,]=0
  for (k in 2:Ntx){  ## Ntx is total number of treatments
    d[k,1:Nbasis] ~ dmnorm(mean_d[],prec_d[,])
  }
  corr ~ dunif(0,0.95)
}
```
