# Supplementary material for: Bayesian splines versus fractional polynomials in network meta-analysis
Source: BMC Med Res Methodol. 2020 Oct 20;20:261. doi: 10.1186/s12874-020-01113-9 (PMC7574305; doi:10.1186/s12874-020-01113-9)
Supplement: Supplementary file 4 — Additional file 4 R code to generate simulation scenario data. [file 12874_2020_1113_MOESM4_ESM.pdf]

Additional File 4

```
# R code to generate data of Simulation Study Section of
#"Bayesian splines versus fractional polynomials in network meta-analysis"
# by A. Heinecke, M. Tallarita, M. De Iorio

# Ns - total number of studies
# Ntx - total number of treatments
# N - total number of records (= nObs in JAGS code for the B-spline model)
# s - vector of length N representing study each time point belongs to
# a - vector of length N represent treatment each time point belongs to
# timen - vector of length N representing each time point
# na - vector of length Ns indicating number of treatments in each study
# tt - matrix of dimensions Ns times maximum number of treatments in the studies,
# representing the treatments included in each study
# se - vector of standard deviations of length N
# I_se - binary vector of length N indicating whether sd is known or unknown
# n - vector of length N representing number of patients

######################################################################
## Initializations for all but the last (non-closed network) scenarios
######################################################################

Ns=3
Ntx=3
N=20
s1<-rep(1,8)
s2<-rep(2,6)
s3<-rep(3,6)
s<-c(s1,s2,s3)
a<-c(rep(1,4),rep(2,4),rep(1,3),rep(2,3),rep(1,3),rep(2,3))
timen<-c(4,8,12,24,4,8,12,24,4,12,24,4,12,24,4,8,12,4,8,12)
na<-c(2,2,2)
tt=matrix(c(1,2,1,3,2,3),nrow=Ns,ncol=2,byrow = TRUE)

n1=100
n2=120
n3=130
n<-c(rep(n1,8),rep(n2,6),rep(n3,6))
tau1=1 #tau in "Simulation study" section
tau2=2
tau3=4

sigma1=sqrt(tau1/n1)
sigma2=sqrt(tau2/n2)
sigma3=sqrt(tau3/n3)
se<-c(rep(sigma1,8),rep(sigma2,6),rep(sigma3,6))
I_se<-rep(1,N)
```

```
theta<-vector() # theta represents alpha_s in "Simulation study" section
for( i in 1:Ns){
  theta[i]=rnorm(1,10)
}

cfb_real=vector() #length N vector of mean change from baseline, Y_sjt in "Simulation study" section

#################################################################
############# Linear scenario
################################################################
time=c(4,8,12,24)
mu11<-matrix(NA,nrow=n1,ncol=4)
y11<-matrix(NA,nrow=n1,ncol=4)
for (j in 1:n1){
  for(t in 1:4){
    mu11[j,t]=theta[1]-1/4*time[t] # represents (gamma_jt + alpha_s) in the section "Simulation study"
    mean_y=mu11[j,t]
    y11[j,t]=rnorm(1,mean_y,sigma1)
  }
}
cfb_real[1]=mean(y11[,1])
cfb_real[2]=mean(y11[,2])
cfb_real[3]=mean(y11[,3])
cfb_real[4]=mean(y11[,4])

mu12<-matrix(NA,nrow=n1,ncol=4)
y12<-matrix(NA,nrow=n1,ncol=4)
for (j in 1:n1){
  for(t in 1:4){
    mu12[j,t]=theta[1]-1/2*time[t]
    mean_y=mu12[j,t]
    y12[j,t]=rnorm(1,mean_y,sigma1)
  }
}
cfb_real[5]=mean(y12[,1])
cfb_real[6]=mean(y12[,2])
cfb_real[7]=mean(y12[,3])
cfb_real[8]=mean(y12[,4])

time=c(4,12,24)
mu21<-matrix(NA,nrow=n2,ncol=3)
y21<-matrix(NA,nrow=n2,ncol=3)
for (j in 1:n2){
  for(t in 1:3){
    mu21[j,t]=theta[2]-1/4*time[t]
    mean_y=mu21[j,t]
    y21[j,t]=rnorm(1,mean_y,sigma2)
  }
}
```

```
cfb_real[9]=mean(y21[,1])
cfb_real[10]=mean(y21[,2])
cfb_real[11]=mean(y21[,3])

mu23<-matrix(NA,nrow=n2,ncol=3)
y23<-matrix(NA,nrow=n2,ncol=3)
for (j in 1:n2){
  for(t in 1:3){
    mu23[j,t]=theta[2]-1/3*time[t]
    mean_y=mu23[j,t]
    y23[j,t]=rnorm(1,mean_y,sigma2)
  }
}
cfb_real[12]=mean(y23[,1])
cfb_real[13]=mean(y23[,2])
cfb_real[14]=mean(y23[,3])

time=c(4,8,12)
mu32<-matrix(NA,nrow=n3,ncol=3)
y32<-matrix(NA,nrow=n3,ncol=3)
for (j in 1:n3){
  for(t in 1:3){
    mu32[j,t]=theta[3]-1/2*time[t]
    mean_y=mu32[j,t]
    y32[j,t]=rnorm(1,mean_y,sigma3)
  }
}
cfb_real[15]=mean(y32[,1])
cfb_real[16]=mean(y32[,2])
cfb_real[17]=mean(y32[,3])

mu33<-matrix(NA,nrow=n3,ncol=3)
y33<-matrix(NA,nrow=n3,ncol=3)
for (j in 1:n3){
  for(t in 1:3){
    mu33[j,t]=theta[3]-1/3*time[t]
    mean_y=mu33[j,t]
    y33[j,t]=rnorm(1,mean_y,sigma3)
  }
}
cfb_real[18]=mean(y33[,1])
cfb_real[19]=mean(y33[,2])
cfb_real[20]=mean(y33[,3])

########################################################
############# Logarithmic scenario
######################################################
time=c(4,8,12,24)
mu11<-matrix(NA,nrow=n1,ncol=4)
```

```
y11<-matrix(NA,nrow=n1,ncol=4)
for (j in 1:n1){
  for(t in 1:4){
    mu11[j,t]=theta[1]-log(time[t])
    mean_y=mu11[j,t]
    y11[j,t]=rnorm(1,mean_y,sigma1)
  }
}
cfb_real[1]=mean(y11[,1])
cfb_real[2]=mean(y11[,2])
cfb_real[3]=mean(y11[,3])
cfb_real[4]=mean(y11[,4])

mu12<-matrix(NA,nrow=n1,ncol=4)
y12<-matrix(NA,nrow=n1,ncol=4)
for (j in 1:n1){
  for(t in 1:4){
    mu12[j,t]=theta[1]-log(time[t])*3
    mean_y=mu12[j,t]
    y12[j,t]=rnorm(1,mean_y,sigma1)
  }
}
cfb_real[5]=mean(y12[,1])
cfb_real[6]=mean(y12[,2])
cfb_real[7]=mean(y12[,3])
cfb_real[8]=mean(y12[,4])

time=c(4,12,24)
mu21<-matrix(NA,nrow=n2,ncol=3)
y21<-matrix(NA,nrow=n2,ncol=3)
for (j in 1:n2){
  for(t in 1:3){
    mu21[j,t]=theta[2]-log(time[t])
    mean_y=mu21[j,t]
    y21[j,t]=rnorm(1,mean_y,sigma2)
  }
}
cfb_real[9]=mean(y21[,1])
cfb_real[10]=mean(y21[,2])
cfb_real[11]=mean(y21[,3])

mu23<-matrix(NA,nrow=n2,ncol=3)
y23<-matrix(NA,nrow=n2,ncol=3)
for (j in 1:n2){
  for(t in 1:3){
    mu23[j,t]=theta[2]-log(time[t])*2
    mean_y=mu23[j,t]
    y23[j,t]=rnorm(1,mean_y,sigma2)
  }
```

```
}
cfb_real[12]=mean(y23[,1])
cfb_real[13]=mean(y23[,2])
cfb_real[14]=mean(y23[,3])

time=c(4,8,12)
mu32<-matrix(NA,nrow=n3,ncol=3)
y32<-matrix(NA,nrow=n3,ncol=3)
for (j in 1:n3){
  for(t in 1:3){
    mu32[j,t]=theta[3]-log(time[t])*3
    mean_y=mu32[j,t]
    y32[j,t]=rnorm(1,mean_y,sigma3)
  }
}
cfb_real[15]=mean(y32[,1])
cfb_real[16]=mean(y32[,2])
cfb_real[17]=mean(y32[,3])


mu33<-matrix(NA,nrow=n3,ncol=3)
y33<-matrix(NA,nrow=n3,ncol=3)
for (j in 1:n3){
  for(t in 1:3){
    mu33[j,t]=theta[3]-log(time[t])*2
    mean_y=mu33[j,t]
    y33[j,t]=rnorm(1,mean_y,sigma3)
  }
}
cfb_real[18]=mean(y33[,1])
cfb_real[19]=mean(y33[,2])
cfb_real[20]=mean(y33[,3])

################################################################
############# piecewise linear monotonic scenario
###############################################################
time=c(4,8,12,24)
timey=c(-3,-5,-6,-6)
mu11<-matrix(NA,nrow=n1,ncol=4)
y11<-matrix(NA,nrow=n1,ncol=4)

for (j in 1:n1){
  for(t in 1:4){
    mu11[j,t]=theta[1]+timey[t]
    mean_y=mu11[j,t]
    y11[j,t]=rnorm(1,mean_y,sigma1)
  }
}
cfb_real[1]=mean(y11[,1])
```

```
cfb_real[2]=mean(y11[,2])
cfb_real[3]=mean(y11[,3])
cfb_real[4]=mean(y11[,4])
timey=c(-6,-7.75,-9,-10)
mu12<-matrix(NA,nrow=n1,ncol=4)
y12<-matrix(NA,nrow=n1,ncol=4)

for (j in 1:n1){
  for(t in 1:4){
    mu12[j,t]=theta[1]+timey[t]
    mean_y=mu12[j,t]
    y12[j,t]=rnorm(1,mean_y,sigma1)
  }
}
cfb_real[5]=mean(y12[,1])
cfb_real[6]=mean(y12[,2])
cfb_real[7]=mean(y12[,3])
cfb_real[8]=mean(y12[,4])

time=c(4,12,24)
timey=c(-3,-6,-6)
mu21<-matrix(NA,nrow=n2,ncol=3)
y21<-matrix(NA,nrow=n2,ncol=3)

for (j in 1:n2){
  for(t in 1:3){
    mu21[j,t]=theta[2]+timey[t]
    mean_y=mu21[j,t]
    y21[j,t]=rnorm(1,mean_y,sigma2)
  }
}
cfb_real[9]=mean(y21[,1])
cfb_real[10]=mean(y21[,2])
cfb_real[11]=mean(y21[,3])

timey=c(-4,-8,-9)
mu23<-matrix(NA,nrow=n2,ncol=3)
y23<-matrix(NA,nrow=n2,ncol=3)

for (j in 1:n2){
  for(t in 1:3){
    mu23[j,t]=theta[2]+timey[t]
    mean_y=mu23[j,t]
    y23[j,t]=rnorm(1,mean_y,sigma2)
  }
}
cfb_real[12]=mean(y23[,1])
cfb_real[13]=mean(y23[,2])
cfb_real[14]=mean(y23[,3])
```

```
time=c(4,8,12)
timey=c(-6,-7.75,-9)
mu32<-matrix(NA,nrow=n3,ncol=3)
y32<-matrix(NA,nrow=n3,ncol=3)

for (j in 1:n3){
  for(t in 1:3){
    mu32[j,t]=theta[3]+timey[t]
    mean_y=mu32[j,t]
    y32[j,t]=rnorm(1,mean_y,sigma3)
  }
}
cfb_real[15]=mean(y32[,1])
cfb_real[16]=mean(y32[,2])
cfb_real[17]=mean(y32[,3])

timey=c(-4,-7,-8)
mu33<-matrix(NA,nrow=n3,ncol=3)
y33<-matrix(NA,nrow=n3,ncol=3)

for (j in 1:n3){
  for(t in 1:3){
    mu33[j,t]=theta[3]+timey[t]
    mean_y=mu33[j,t]
    y33[j,t]=rnorm(1,mean_y,sigma3)
  }
}
cfb_real[18]=mean(y33[,1])
cfb_real[19]=mean(y33[,2])
cfb_real[20]=mean(y33[,3])

############################################################
############# piecewise linear scenario with binary outcomes
########################################################
time=c(4,8,12,24)
timey=c(-3,-5,-6,-6)
mu11<-matrix(NA,nrow=n1,ncol=4)
y11<-matrix(NA,nrow=n1,ncol=4)

for (j in 1:n1){
  for(t in 1:4){
    mu11[j,t]=theta[1]+timey[t]
    mean_y=mu11[j,t]
    y11[j,t]=rnorm(1,mean_y,sigma1)
  }
}
cfb_real[1]=mean(y11[,1])
cfb_real[2]=mean(y11[,2])
```

```
cfb_real[3]=mean(y11[,3])
cfb_real[4]=mean(y11[,4])
timey=c(-6,-7.75,-9,-10)
mu12<-matrix(NA,nrow=n1,ncol=4)
y12<-matrix(NA,nrow=n1,ncol=4)

for (j in 1:n1){
  for(t in 1:4){
    mu12[j,t]=theta[1]+timey[t]
    mean_y=mu12[j,t]
    y12[j,t]=rnorm(1,mean_y,sigma1)
  }
}
cfb_real[5]=mean(y12[,1])
cfb_real[6]=mean(y12[,2])
cfb_real[7]=mean(y12[,3])
cfb_real[8]=mean(y12[,4])

time=c(4,12,24)
timey=c(-3,-6,-6)
mu21<-matrix(NA,nrow=n2,ncol=3)
y21<-matrix(NA,nrow=n2,ncol=3)

for (j in 1:n2){
  for(t in 1:3){
    mu21[j,t]=theta[2]+timey[t]
    mean_y=mu21[j,t]
    y21[j,t]=rnorm(1,mean_y,sigma2)
  }
}
cfb_real[9]=mean(y21[,1])
cfb_real[10]=mean(y21[,2])
cfb_real[11]=mean(y21[,3])

timey=c(-4,-8,-9)
mu23<-matrix(NA,nrow=n2,ncol=3)
y23<-matrix(NA,nrow=n2,ncol=3)

for (j in 1:n2){
  for(t in 1:3){
    mu23[j,t]=theta[2]+timey[t]
    mean_y=mu23[j,t]
    y23[j,t]=rnorm(1,mean_y,sigma2)
  }
}
cfb_real[12]=mean(y23[,1])
cfb_real[13]=mean(y23[,2])
cfb_real[14]=mean(y23[,3])
```

```
time=c(4,8,12)
timey=c(-6,-7.75,-9)
mu32<-matrix(NA,nrow=n3,ncol=3)
y32<-matrix(NA,nrow=n3,ncol=3)

for (j in 1:n3){
  for(t in 1:3){
    mu32[j,t]=theta[3]+timey[t]
    mean_y=mu32[j,t]
    y32[j,t]=rnorm(1,mean_y,sigma3)
  }
}
cfb_real[15]=mean(y32[,1])
cfb_real[16]=mean(y32[,2])
cfb_real[17]=mean(y32[,3])

timey=c(-4,-7,-8)
mu33<-matrix(NA,nrow=n3,ncol=3)
y33<-matrix(NA,nrow=n3,ncol=3)

for (j in 1:n3){
  for(t in 1:3){
    mu33[j,t]=theta[3]+timey[t]
    mean_y=mu33[j,t]
    y33[j,t]=rnorm(1,mean_y,sigma3)
  }
}
cfb_real[18]=mean(y33[,1])
cfb_real[19]=mean(y33[,2])
cfb_real[20]=mean(y33[,3])

cfb<-cfb_real

cs<-(cfb-mean(cfb))/sd(cfb)

#logit
prob<-exp(1-cs)/(1+exp(1-cs))


cfb_real<-vector()
for(i in 1:N){
  cfb_real[i]<-rbinom(1,n,prob[i])
}

############################################################
############# mixed scenario
##########################################################
time=c(4,8,12,24)
timey=c(0,0,-2.25,-8)
```

```
mu11<-matrix(NA,nrow=n1,ncol=4)
y11<-matrix(NA,nrow=n1,ncol=4)

for (j in 1:n1){
  for(t in 1:4){
    mu11[j,t]=theta[1]+timey[t]
    mean_y=mu11[j,t]
    y11[j,t]=rnorm(1,mean_y,sigma1)
  }
}
cfb_real[1]=mean(y11[,1])
cfb_real[2]=mean(y11[,2])
cfb_real[3]=mean(y11[,3])
cfb_real[4]=mean(y11[,4])

mu12<-matrix(NA,nrow=n1,ncol=4)
y12<-matrix(NA,nrow=n1,ncol=4)

timey=c(0,0,0,0)
for (j in 1:n1){
  for(t in 1:4){
    mu12[j,t]=theta[1]+timey[t]
    mean_y=mu12[j,t]
    y12[j,t]=rnorm(1,mean_y,sigma1)
  }
}
cfb_real[5]=mean(y12[,1])
cfb_real[6]=mean(y12[,2])
cfb_real[7]=mean(y12[,3])
cfb_real[8]=mean(y12[,4])

time=c(4,12,24)
mu21<-matrix(NA,nrow=n2,ncol=3)
y21<-matrix(NA,nrow=n2,ncol=3)

timey=c(0,-2.25,-8)
for (j in 1:n2){
  for(t in 1:3){
    mu21[j,t]=theta[2]+timey[t]
    mean_y=mu21[j,t]
    y21[j,t]=rnorm(1,mean_y,sigma2)
  }
}
cfb_real[9]=mean(y21[,1])
cfb_real[10]=mean(y21[,2])
cfb_real[11]=mean(y21[,3])

mu23<-matrix(NA,nrow=n2,ncol=3)
y23<-matrix(NA,nrow=n2,ncol=3)
```

```
for (j in 1:n2){
  for(t in 1:3){
    mu23[j,t]=theta[2]+(-time[t]^2-25*time[t])/100
    mean_y=mu23[j,t]
    y23[j,t]=rnorm(1,mean_y,sigma2)
  }
}
cfb_real[12]=mean(y23[,1])
cfb_real[13]=mean(y23[,2])
cfb_real[14]=mean(y23[,3])

time=c(4,8,12)
mu32<-matrix(NA,nrow=n3,ncol=3)
y32<-matrix(NA,nrow=n3,ncol=3)

timey=c(0,0,0, 0,0)
for (j in 1:n3){
  for(t in 1:3){
    mu32[j,t]=theta[3]
    mean_y=mu32[j,t]
    y32[j,t]=rnorm(1,mean_y,sigma3)
  }
}
cfb_real[15]=mean(y32[,1])
cfb_real[16]=mean(y32[,2])
cfb_real[17]=mean(y32[,3])

mu33<-matrix(NA,nrow=n3,ncol=3)
y33<-matrix(NA,nrow=n3,ncol=3)

for (j in 1:n3){
  for(t in 1:3){
    mu33[j,t]=theta[3]+(-time[t]^2-25*time[t])/100
    mean_y=mu33[j,t]
    y33[j,t]=rnorm(1,mean_y,sigma3)
  }
}
cfb_real[18]=mean(y33[,1])
cfb_real[19]=mean(y33[,2])
cfb_real[20]=mean(y33[,3])

###############################################
############# non-monotonic scenario
###############################################
time=c(4,8,12,24)
timeA=c(-3,-8,-10,-4)
timeB=c(-6,-10,-6,-3)
timeC=c(-8,-7,-6,-4)
```

```
mu11<-matrix(NA,nrow=n1,ncol=4)
y11<-matrix(NA,nrow=n1,ncol=4)

for (j in 1:n1){
  for(t in 1:4){
    mu11[j,t]=theta[1]+timeA[t]
    mean_y=mu11[j,t]
    y11[j,t]=rnorm(1,mean_y,sigma1)
  }
}
cfb_real[1]=mean(y11[,1])
cfb_real[2]=mean(y11[,2])
cfb_real[3]=mean(y11[,3])
cfb_real[4]=mean(y11[,4])

mu12<-matrix(NA,nrow=n1,ncol=4)
y12<-matrix(NA,nrow=n1,ncol=4)
timeA=c(-3,-8,-10,-4)
timeB=c(-6,-10,-6,-3)
timeC=c(-8,-7,-6,-4)
for (j in 1:n1){
  for(t in 1:4){
    mu12[j,t]=theta[1]+timeB[t]
    mean_y=mu12[j,t]
    y12[j,t]=rnorm(1,mean_y,sigma1)
  }
}
cfb_real[5]=mean(y12[,1])
cfb_real[6]=mean(y12[,2])
cfb_real[7]=mean(y12[,3])
cfb_real[8]=mean(y12[,4])

time=c(4,12,24)
mu21<-matrix(NA,nrow=n2,ncol=3)
y21<-matrix(NA,nrow=n2,ncol=3)
timeA=c(-3,-10,-4)
timeB=c(-6,-6,-3)
timeC=c(-8,-6,-4)
for (j in 1:n2){
  for(t in 1:3){
    mu21[j,t]=theta[2]+timeA[t]
    mean_y=mu21[j,t]
    y21[j,t]=rnorm(1,mean_y,sigma2)
  }
}
cfb_real[9]=mean(y21[,1])
cfb_real[10]=mean(y21[,2])
cfb_real[11]=mean(y21[,3])
```

```
mu23<-matrix(NA,nrow=n2,ncol=3)
y23<-matrix(NA,nrow=n2,ncol=3)
timeA=c(-3,-10,-4)
timeB=c(-6,-6,-3)
timeC=c(-8,-6,-4)
for (j in 1:n2){
  for(t in 1:3){
    mu23[j,t]=theta[2]+timeC[t]
    mean_y=mu23[j,t]
    y23[j,t]=rnorm(1,mean_y,sigma2)
  }
}
cfb_real[12]=mean(y23[,1])
cfb_real[13]=mean(y23[,2])
cfb_real[14]=mean(y23[,3])

time=c(4,8,12)
timeA=c(-3,-8,-10)
timeB=c(-6,-10,-6)
timeC=c(-8,-7,-6)
mu32<-matrix(NA,nrow=n3,ncol=3)
y32<-matrix(NA,nrow=n3,ncol=3)

for (j in 1:n3){
  for(t in 1:3){
    mu32[j,t]=theta[3]+timeB[t]
    mean_y=mu32[j,t]
    y32[j,t]=rnorm(1,mean_y,sigma3)
  }
}
cfb_real[15]=mean(y32[,1])
cfb_real[16]=mean(y32[,2])
cfb_real[17]=mean(y32[,3])

mu33<-matrix(NA,nrow=n3,ncol=3)
y33<-matrix(NA,nrow=n3,ncol=3)
timeA=c(-3,-8,-10)
timeB=c(-6,-10,-6)
timeC=c(-8,-7,-6)
for (j in 1:n3){
  for(t in 1:3){
    mu33[j,t]=theta[3]+timeC[t]
    mean_y=mu33[j,t]
    y33[j,t]=rnorm(1,mean_y,sigma3)
  }
}
cfb_real[18]=mean(y33[,1])
cfb_real[19]=mean(y33[,2])
cfb_real[20]=mean(y33[,3])
```

```
#####################################################
############# non-monotonic scenario with binary outcomes
######################################################
time=c(4,8,12,24)
timeA=c(-3,-8,-10,-4)
timeB=c(-6,-10,-6,-3)
timeC=c(-8,-7,-6,-4)
mu11<-matrix(NA,nrow=n1,ncol=4)
y11<-matrix(NA,nrow=n1,ncol=4)

for (j in 1:n1){
  for(t in 1:4){
    mu11[j,t]=theta[1]+timeA[t]
    mean_y=mu11[j,t]
    y11[j,t]=rnorm(1,mean_y,sigma1)
  }
}
cfb_real[1]=mean(y11[,1])
cfb_real[2]=mean(y11[,2])
cfb_real[3]=mean(y11[,3])
cfb_real[4]=mean(y11[,4])

mu12<-matrix(NA,nrow=n1,ncol=4)
y12<-matrix(NA,nrow=n1,ncol=4)
timeA=c(-3,-8,-10,-4)
timeB=c(-6,-10,-6,-3)
timeC=c(-8,-7,-6,-4)
for (j in 1:n1){
  for(t in 1:4){
    mu12[j,t]=theta[1]+timeB[t]
    mean_y=mu12[j,t]
    y12[j,t]=rnorm(1,mean_y,sigma1)
  }
}
cfb_real[5]=mean(y12[,1])
cfb_real[6]=mean(y12[,2])
cfb_real[7]=mean(y12[,3])
cfb_real[8]=mean(y12[,4])

time=c(4,12,24)
mu21<-matrix(NA,nrow=n2,ncol=3)
y21<-matrix(NA,nrow=n2,ncol=3)
timeA=c(-3,-10,-4)
timeB=c(-6,-6,-3)
timeC=c(-8,-6,-4)
for (j in 1:n2){
  for(t in 1:3){
    mu21[j,t]=theta[2]+timeA[t]
```

```
    mean_y=mu21[j,t]
    y21[j,t]=rnorm(1,mean_y,sigma2)
  }
}
cfb_real[9]=mean(y21[,1])
cfb_real[10]=mean(y21[,2])
cfb_real[11]=mean(y21[,3])

mu23<-matrix(NA,nrow=n2,ncol=3)
y23<-matrix(NA,nrow=n2,ncol=3)
timeA=c(-3,-10,-4)
timeB=c(-6,-6,-3)
timeC=c(-8,-6,-4)
for (j in 1:n2){
  for(t in 1:3){
    mu23[j,t]=theta[2]+timeC[t]
    mean_y=mu23[j,t]
    y23[j,t]=rnorm(1,mean_y,sigma2)
  }
}
cfb_real[12]=mean(y23[,1])
cfb_real[13]=mean(y23[,2])
cfb_real[14]=mean(y23[,3])

time=c(4,8,12)
timeA=c(-3,-8,-10)
timeB=c(-6,-10,-6)
timeC=c(-8,-7,-6)
mu32<-matrix(NA,nrow=n3,ncol=3)
y32<-matrix(NA,nrow=n3,ncol=3)

for (j in 1:n3){
  for(t in 1:3){
    mu32[j,t]=theta[3]+timeB[t]
    mean_y=mu32[j,t]
    y32[j,t]=rnorm(1,mean_y,sigma3)
  }
}
cfb_real[15]=mean(y32[,1])
cfb_real[16]=mean(y32[,2])
cfb_real[17]=mean(y32[,3])

mu33<-matrix(NA,nrow=n3,ncol=3)
y33<-matrix(NA,nrow=n3,ncol=3)
timeA=c(-3,-8,-10)
timeB=c(-6,-10,-6)
timeC=c(-8,-7,-6)
for (j in 1:n3){
  for(t in 1:3){
```

```
    mu33[j,t]=theta[3]+timeC[t]
    mean_y=mu33[j,t]
    y33[j,t]=rnorm(1,mean_y,sigma3)
  }
}
cfb_real[18]=mean(y33[,1])
cfb_real[19]=mean(y33[,2])
cfb_real[20]=mean(y33[,3])

cfb<-cfb_real

cs<-(cfb-mean(cfb))/sd(cfb)

#logit
prob<-exp(1-cs)/(1+exp(1-cs))


cfb_real<-vector()
for(i in 1:N){
  cfb_real[i]<-rbinom(1,n,prob[i])
}

##########################################################
############# MTC scenario
##########################################################
Ns=3
Ntx=3
N=20
s1<-rep(1,8)
s2<-rep(2,6)
s3<-rep(3,6)
s<-c(s1,s2,s3)
a<-c(rep(1,4),rep(2,4),rep(1,3),rep(2,3),rep(1,3),rep(2,3))
timen<-c(4,8,12,24,4,8,12,24,4,12,24,4,12,24,4,8,12,4,8,12)
na<-c(2,2,2)
tt=matrix(c(1,2,1,3,2,3),nrow=Ns,ncol=2,byrow = TRUE)

n1=100
n2=120
n3=130
n<-c(rep(n1,8),rep(n2,6),rep(n3,6))

sd=1.2
sigma1=sd/sqrt(n1)
sigma2=sd/sqrt(n2)
sigma3=sd/sqrt(n3)
se<-c(rep(sigma1,8),rep(sigma2,6),rep(sigma3,6))
I_se<-rep(1,N)
```

```
cfb_real=vector() #is the vector of mean change from baseline of length N,
#Y_sjt in the section "Simulation study"

mu11<-matrix(NA,nrow=n1,ncol=4)
y11<-matrix(NA,nrow=n1,ncol=4)

for (j in 1:n1){
  for(t in 1:4){
    mu=rnorm(1, -1,1)
    delta=rnorm(1, 0.5,1)
    mu11[j,t]=mu+delta
    mean_y=mu11[j,t]
    y11[j,t]=rnorm(1,mean_y,sigma1^2)
  }
}
cfb_real[1]=mean(y11[,1])
cfb_real[2]=mean(y11[,2])
cfb_real[3]=mean(y11[,3])
cfb_real[4]=mean(y11[,4])

mu12<-matrix(NA,nrow=n1,ncol=4)
y12<-matrix(NA,nrow=n1,ncol=4)
for (j in 1:n1){
  for(t in 1:4){
    mu=rnorm(1, -1,1)
    delta=rnorm(1, 0.5,1)
    mu12[j,t]=mu+delta
    mean_y=mu12[j,t]
    y12[j,t]=rnorm(1,mean_y,sigma1^2)
  }
}
cfb_real[5]=mean(y12[,1])
cfb_real[6]=mean(y12[,2])
cfb_real[7]=mean(y12[,3])
cfb_real[8]=mean(y12[,4])

time=c(4,12,24)
mu21<-matrix(NA,nrow=n2,ncol=3)
y21<-matrix(NA,nrow=n2,ncol=3)
for (j in 1:n2){
  for(t in 1:3){
    mu=rnorm(1, -2,1)
    delta=rnorm(1, 0.5,1)
    mu21[j,t]=mu+delta
    mean_y=mu21[j,t]
    y21[j,t]=rnorm(1,mean_y,sigma2^2)
  }
}
cfb_real[9]=mean(y21[,1])
```

```
cfb_real[10]=mean(y21[,2])
cfb_real[11]=mean(y21[,3])

mu23<-matrix(NA,nrow=n2,ncol=3)
y23<-matrix(NA,nrow=n2,ncol=3)
for (j in 1:n2){
  for(t in 1:3){
    mu=rnorm(1, -2,1)
    delta=rnorm(1, 0.5,1)
    mu23[j,t]=mu+delta
    mean_y=mu23[j,t]
    y23[j,t]=rnorm(1,mean_y,sigma2^2)
  }
}
cfb_real[12]=mean(y23[,1])
cfb_real[13]=mean(y23[,2])
cfb_real[14]=mean(y23[,3])

time=c(4,8,12)
mu32<-matrix(NA,nrow=n3,ncol=3)
y32<-matrix(NA,nrow=n3,ncol=3)

for (j in 1:n3){
  for(t in 1:3){
    mu=rnorm(1, -3,1)
    delta=rnorm(1, 0.5,1)
    mu32[j,t]=mu+delta
    mean_y=mu32[j,t]
    y32[j,t]=rnorm(1,mean_y,sigma3^2)
  }
}
cfb_real[15]=mean(y32[,1])
cfb_real[16]=mean(y32[,2])
cfb_real[17]=mean(y32[,3])

mu33<-matrix(NA,nrow=n3,ncol=3)
y33<-matrix(NA,nrow=n3,ncol=3)
for (j in 1:n3){
  for(t in 1:3){
    mu=rnorm(1, -3,1)
    delta=rnorm(1, 0.5,1)
    mu33[j,t]=mu+delta
    mean_y=mu33[j,t]
    y33[j,t]=rnorm(1,mean_y,sigma3^2)
  }
}
cfb_real[18]=mean(y33[,1])
cfb_real[19]=mean(y33[,2])
cfb_real[20]=mean(y33[,3])
```

```
############################################################
############# BEST-ITP scenario
###########################################################
y11<-matrix(NA,nrow=n1,ncol=4)
time=c(4,8,12,24)
for (j in 1:n1){
  for(t in 1:4){
    mean_y=(phi[1]+delta[1])*(1-exp(p[1]*time[t]))/(1-exp(p[1]*24))
    sigma11=(1-exp(p[1]*time[t]))/(1-exp(p[1]*24))*1.2/sqrt(n1)
    y11[j,t]=rnorm(1,mean_y,sigma11^2)
  }
}
cfb_real[1]=mean(y11[,1])
cfb_real[2]=mean(y11[,2])
cfb_real[3]=mean(y11[,3])
cfb_real[4]=mean(y11[,4])

y12<-matrix(NA,nrow=n1,ncol=4)
for (j in 1:n1){
  for(t in 1:4){
    mean_y=(phi[1]+delta[2])*(1-exp(p[2]*time[t]))/(1-exp(p[2]*24))
    sigma12=((1-exp(p[2]*time[t]))/(1-exp(p[2]*24))*1.2/sqrt(n1))
    y12[j,t]=rnorm(1,mean_y,sigma12^2)
  }
}
cfb_real[5]=mean(y12[,1])
cfb_real[6]=mean(y12[,2])
cfb_real[7]=mean(y12[,3])
cfb_real[8]=mean(y12[,4])

time=c(4,12,24)
y21<-matrix(NA,nrow=n2,ncol=3)
for (j in 1:n2){
  for(t in 1:3){
    mean_y=(phi[2]+delta[1])*(1-exp(p[1]*time[t]))/(1-exp(p[1]*24))
    sigma21=((1-exp(p[1]*time[t]))/(1-exp(p[1]*24))*1.2/sqrt(n2))
    y21[j,t]=rnorm(1,mean_y,sigma21^2)
  }
}
cfb_real[9]=mean(y21[,1])
cfb_real[10]=mean(y21[,2])
cfb_real[11]=mean(y21[,3])

y23<-matrix(NA,nrow=n2,ncol=3)
for (j in 1:n2){
  for(t in 1:3){
    mean_y=(phi[2]+delta[3])*(1-exp(p[3]*time[t]))/(1-exp(p[3]*24))
    sigma22=((1-exp(p[3]*time[t]))/(1-exp(p[3]*24))*1.2/sqrt(n2))
```

```
    y23[j,t]=rnorm(1,mean_y,sigma22^2)
  }
}
cfb_real[12]=mean(y23[,1])
cfb_real[13]=mean(y23[,2])
cfb_real[14]=mean(y23[,3])

time=c(4,8,12)
y32<-matrix(NA,nrow=n3,ncol=3)

for (j in 1:n3){
  for(t in 1:3){
    mean_y=(phi[3]+delta[2])*(1-exp(p[2]*time[t]))/(1-exp(p[2]*24))
    sigma31=((1-exp(p[2]*time[t]))/(1-exp(p[2]*24))*1.2/sqrt(n3))
    y32[j,t]=rnorm(1,mean_y,sigma31^2)
  }
}
cfb_real[15]=mean(y32[,1])
cfb_real[16]=mean(y32[,2])
cfb_real[17]=mean(y32[,3])

y33<-matrix(NA,nrow=n3,ncol=3)
for (j in 1:n3){
  for(t in 1:3){
    mean_y=(phi[3]+delta[3])*(1-exp(p[3]*time[t]))/(1-exp(p[3]*24))
    sigma32=((1-exp(p[3]*time[t]))/(1-exp(p[3]*24))*1.2/sqrt(n3))
    y33[j,t]=rnorm(1,mean_y,sigma32^2)
  }
}
cfb_real[18]=mean(y33[,1])
cfb_real[19]=mean(y33[,2])
cfb_real[20]=mean(y33[,3])

se<-c(rep(sigma11,4),rep(sigma12,4),rep(sigma21,3),rep(sigma22,3),rep(sigma31,3),rep(sigma32,3))

#################################################################
############# piecewise linear scenario under the non-closed network scenario
#################################################################
Ns=4
Ntx=4
N=28
s1<-rep(1,8)
s2<-rep(2,6)
s3<-rep(3,6)
s4<-rep(4,8)
s<-c(s1,s2,s3,s4)
a<-c(rep(1,4),rep(2,4),rep(1,3),rep(2,3),rep(1,3),rep(2,3),rep(1,4),rep(2,4))
timen<-c(4,8,12,24,4,8,12,24,4,12,24,4,12,24,4,8,12,4,8,12,4,8,12,24,4,8,12,24)
na<-c(2,2,2,2)
```

```
tt=matrix(c(1,2,1,3,2,3,2,4),nrow=4,ncol=2,byrow = TRUE)

n1=100
n2=120
n3=130
n4=110
n<-c(rep(n1,8),rep(n2,6),rep(n3,6),rep(n4,8))
tau1=1 #tau is tau in the section "Simulation study"
tau2=2
tau3=4
tau4=3

sigma1=sqrt(tau1/n1)
sigma2=sqrt(tau2/n2)
sigma3=sqrt(tau3/n3)
sigma4=sqrt(tau4/n4)
se<-c(rep(sigma1,8),rep(sigma2,6),rep(sigma3,6),rep(sigma4,8))
I_se<-rep(1,N)

theta<-vector() # theta represents alpha_s in the section "Simulation study"
for( i in 1:Ns){
  theta[i]=rnorm(1,10)
}

cfb_real=vector()
time=c(4,8,12,24)
timey=c(-3,-5,-6,-6)
mu11<-matrix(NA,nrow=n1,ncol=4)
y11<-matrix(NA,nrow=n1,ncol=4)
for (j in 1:n1){
  for(t in 1:4){
    mu11[j,t]=theta[1]+timey[t]
    mean_y=mu11[j,t]
    y11[j,t]=rnorm(1,mean_y,sigma1)
  }
}
cfb_real[1]=mean(y11[,1])
cfb_real[2]=mean(y11[,2])
cfb_real[3]=mean(y11[,3])
cfb_real[4]=mean(y11[,4])

timey=c(-6,-7.75,-9,-10)
mu12<-matrix(NA,nrow=n1,ncol=4)
y12<-matrix(NA,nrow=n1,ncol=4)

for (j in 1:n1){
  for(t in 1:4){
    mu12[j,t]=theta[1]+timey[t]
    mean_y=mu12[j,t]
```

```
    y12[j,t]=rnorm(1,mean_y,sigma1)
  }
}
cfb_real[5]=mean(y12[,1])
cfb_real[6]=mean(y12[,2])
cfb_real[7]=mean(y12[,3])
cfb_real[8]=mean(y12[,4])

time=c(4,12,24)
timey=c(-3,-6,-6)
mu21<-matrix(NA,nrow=n2,ncol=3)
y21<-matrix(NA,nrow=n2,ncol=3)
sigma2=sqrt(2)
for (j in 1:n2){
  for(t in 1:3){
    mu21[j,t]=theta[2]+timey[t]
    mean_y=mu21[j,t]
    y21[j,t]=rnorm(1,mean_y,sigma2)
  }
}
cfb_real[9]=mean(y21[,1])
cfb_real[10]=mean(y21[,2])
cfb_real[11]=mean(y21[,3])

timey=c(-4,-8,-9)
mu23<-matrix(NA,nrow=n2,ncol=3)
y23<-matrix(NA,nrow=n2,ncol=3)
for (j in 1:n2){
  for(t in 1:3){
    mu23[j,t]=theta[2]+timey[t]
    mean_y=mu23[j,t]
    y23[j,t]=rnorm(1,mean_y,sigma2)
  }
}
cfb_real[12]=mean(y23[,1])
cfb_real[13]=mean(y23[,2])
cfb_real[14]=mean(y23[,3])

time=c(4,8,12)
timey=c(-6,-7.75,-9)
mu32<-matrix(NA,nrow=n3,ncol=3)
y32<-matrix(NA,nrow=n3,ncol=3)
for (j in 1:n3){
  for(t in 1:3){
    mu32[j,t]=theta[3]+timey[t]
    mean_y=mu32[j,t]
    y32[j,t]=rnorm(1,mean_y,sigma3)
  }
}
```

```
cfb_real[15]=mean(y32[,1])
cfb_real[16]=mean(y32[,2])
cfb_real[17]=mean(y32[,3])

timey=c(-4,-7,-8)
mu33<-matrix(NA,nrow=n3,ncol=3)
y33<-matrix(NA,nrow=n3,ncol=3)
for (j in 1:n3){
  for(t in 1:3){
    mu33[j,t]=theta[3]+timey[t]
    mean_y=mu33[j,t]
    y33[j,t]=rnorm(1,mean_y,sigma3)
  }
}
cfb_real[18]=mean(y33[,1])
cfb_real[19]=mean(y33[,2])
cfb_real[20]=mean(y33[,3])

time=c(4,8,12,24)
timey=c(-6,-7.75,-9,-10)
mu42<-matrix(NA,nrow=n4,ncol=4)
y42<-matrix(NA,nrow=n4,ncol=4)
for (j in 1:n4){
  for(t in 1:4){
    mu42[j,t]=theta[4]+timey[t]
    mean_y=mu42[j,t]
    y42[j,t]=rnorm(1,mean_y,sigma4)
  }
}
cfb_real[21]=mean(y11[,1])
cfb_real[22]=mean(y11[,2])
cfb_real[23]=mean(y11[,3])
cfb_real[24]=mean(y11[,4])
timey=c(-2,-3,-4,-6)
mu44<-matrix(NA,nrow=n4,ncol=4)
y44<-matrix(NA,nrow=n4,ncol=4)

for (j in 1:n4){
  for(t in 1:4){
    mu44[j,t]=theta[4]+timey[t]
    mean_y=mu44[j,t]
    y44[j,t]=rnorm(1,mean_y,sigma4)
  }
}
cfb_real[25]=mean(y42[,1])
cfb_real[26]=mean(y42[,2])
cfb_real[27]=mean(y42[,3])
cfb_real[28]=mean(y42[,4])
```
